# Development of Disease-Resistance-Associated Microsatellite DNA Markers for Selective Breeding of Tilapia (*Oreochromis* spp.) Farmed in Taiwan

**DOI:** 10.3390/genes13010099

**Published:** 2021-12-31

**Authors:** Che-Chun Chen, Chang-Wen Huang, Chung-Yen Lin, Chia-Hui Ho, Hong Nhat Pham, Te-Hua Hsu, Tzu-Tang Lin, Rong-Hwa Chen, Shuenn-Der Yang, Chin-I. Chang, Hong-Yi Gong

**Affiliations:** 1 Doctoral Degree Program in Marine Biotechnology, National Taiwan Ocean University, Keelung 20224, Taiwan; fassadze@gmail.com (C.-C.C.); cwhuang@mail.ntou.edu.tw (C.-W.H.); 2 Doctoral Degree Program in Marine Biotechnology, Academia Sinica, Taipei 11529, Taiwan; cylin@iis.sinica.edu.tw; 3 Department of Aquaculture, National Taiwan Ocean University, Keelung 20224, Taiwan; gyyuty1@yahoo.com.tw (C.-H.H.); hongnhat@ria1.org (H.N.P.); realgigi@mail.ntou.edu.tw (T.-H.H.); 4 Center of Excellence for the Oceans, National Taiwan Ocean University, Keelung 20224, Taiwan; 5 Institute of Information Science, Academia Sinica, Taipei 11529, Taiwan; tzutang@iis.sinica.edu.tw; 6 Institute of Fisheries Science, National Taiwan University, Taipei 10617, Taiwan; 7 Research Institute for Aquaculture No. 1 (RIA1), Dinh Bang, Tu Son 16352, Bac Ninh, Vietnam; 8 Freshwater Aquaculture Research Center, Fisheries Research Institute, Council of Agriculture, Changhua 50562, Taiwan; rhchen@mail.tfrin.gov.tw (R.-H.C.); sdyang@mail.tfrin.gov.tw (S.-D.Y.); 9 Fisheries Research Institute, Council of Agriculture, Keelung 20246, Taiwan; cichang@mail.tfrin.gov.tw

**Keywords:** tilapia, disease resistance, *Streptococcus iniae*, gene amplification, hepcidin, progranulin, piscidin, microsatellite, marker-assisted selection, predictive model

## Abstract

There are numerous means to improve the tilapia aquaculture industry, and one is to develop disease resistance through selective breeding using molecular markers. In this study, 11 disease-resistance-associated microsatellite markers including 3 markers linked to *hamp2*, 4 linked to *hamp1*, 1 linked to *pgrn2*, 2 linked to *pgrn1*, and 1 linked to *piscidin 4* (*TP4*) genes were established for tilapia strains farmed in Taiwan after challenge with *Streptococcus inae*. The correlation analysis of genotypes and survival revealed a total of 55 genotypes related to survival by the chi-square and Z-test. Although fewer markers were found in B and N2 strains compared with A strain, they performed well in terms of disease resistance. It suggested that this may be due to the low potency of some genotypes and the combinatorial arrangement between them. Therefore, a predictive model was built by the genotypes of the parental generation and the mortality rate of different combinations was calculated. The results show the same trend of predicted mortality in the offspring of three new disease-resistant strains as in the challenge experiment. The present findings is a nonkilling method without requiring the selection by challenge with bacteria or viruses and might increase the possibility of utilization of selective breeding using SSR markers in farms.

## 1. Introduction

Tilapia comprises over 100 species of freshwater fish in Africa that belong to the genera *Oreochromis*, *Sarotherodon*, and *Tilapia*. Seventy-five countries have economies of scale for feeding Nile tilapia. Tilapia is one of the most important economic farmed fish in Taiwan, as well as in the world. According to the Food and Agriculture Organization (FAO), tilapia is one of the important species for providing protein in terms of the human diet [1]. Selective breeding programs have been widely used in aquatic farming (more than 60 species) for fish such as common carp, grass carp, rainbow trout, Atlantic salmon, channel catfish, sea bream, oyster, shrimp, and tilapia [2]. The GIFT strain (Genetic Improvement of Farmed Tilapia) in Nile tilapia (*Oreochromis niloticus*) started in 1989 and originated from the GIFT selective breeding project in the Philippines [3]. While the GIFT strain is well known for its superior growth performance, it is characterized by a comparatively weakened immune system [4].

Diseases associated with Nile tilapia have gathered serious attention in recent years, including viral encephalitis of tilapia larvae (primary characterization of a novel herpes-like virus) and TLEV (tilapia larva encephalitis virus) [5,6]. Many Gram-positive and Gram-negative bacteria can infect tilapia, such as of the following genera: *Aeromonas, Citrobacter, Edwardsiella, Flavobacterium, Pseudomonas, Streptococcus*, and *Mycobacterium* [6,7]. Among these, *Streptococcus agalactiae* and *Streptococcus iniae* are the major bacterial pathogens in tilapia [8]. To solve problems of the related diseases, research has sought to improve the immune capacity of fish through the upregulation expression of antimicrobial peptides (AMPs) [9]. AMP expression is one mechanism used by the innate immune system of fish [10] that can effectively inhibit the growth of bacteria, and even improved survival after the injection of synthetic AMPs has been observed [11]. In fish, most AMPs are α-helical peptides [12]. Moreover, a total of 122 fish AMPs have been reported, including cathelicidin, chryosophsin, dicentracin, epinecidine, hepcidin, misgurin, oncorhynsin, and piscidin [13,14,15,16]. In contrast to blue tilapia (*Oreochromis aureus*), Nile tilapia has stronger disease resistance [17]. Another study also revealed that Nile tilapia had more AMP genes than blue tilapia (*Oreochromis aureus*), especially hepcidin [18]. Additionally, gene diversification and amplification processes have occurred in fish hepcidin [19]. These results suggest a correlation between disease resistance and AMPs. 

Hepcidin is a regulatory factor of iron and also a hepatic antimicrobial peptide (HAMP) that is primarily expressed in the liver [20,21]. It has been extensively studied in various species, including fish. Three hepcidins have been found in *Oreochromis mossambicus*, namely Th1-5, Th2-2, and Th2-3 [21]. Two AMPs, Hep-JF1 and Hep-JF2, were found in Japanese flounder (*Paralichthys olivaceus*). Six hepcidin genes (LcHamps) with diversified regulation and functions involved in antibacterial activity, antiviral activity, and regulation of intracellular iron metabolism were identified in large yellow croaker (*Larimichthys crocea*) genome [19]. Hepcidin showed the strongest expression in the liver after LPS induction on the convict cichlid (*Archocentrus nigrofasciatus*) [11]. Studies of pathogenic bacteria treated with synthetic hepcidin peptides showed that the pathogen cell membranes were destroyed. Studies also indicated that oral intake or injection of hepcidin in zebrafish could inhibit bacterial growth and stimulate the host immune response, which significantly improved their survival rate following pathogen infection [22,23]. Hepcidin was used against *Staphylococcus aureus* and *Vibrio vulnificus* in spotted grouper (*Epinephelus coioides*) [24]. Pagaporn et al. [25] mentioned that hepcidin could inhibit both virulence and growth of bacteria by reducing the level of metals (e.g., Fe) in fish tissues. The study indicated that hepcidin enhanced streptococcosis resistance in Nile tilapia.

Simple sequence repeats (SSRs), also known as microsatellites or short tandem repeats (STRs), are tandem repeats of short sequence motifs commonly found in eukaryotic genomes. They are also considered as useful genetic markers for genetic diversity analysis, DNA fingerprinting, and linkage mapping. Several studies have shown that the distribution of SSRs in the genome is non-random. There are more SSRs in the untranslated regions (UTRs) than in the coding regions and are thought to be associated with the regulation of gene expression [26]. Recent studies have revealed that microsatellite polymorphisms on a genome-wide scale contribute to the heritability of human gene expression. Evidence has been found for the role of microsatellites in regulating transcription factor binding, methylation, promoter, enhancers, mRNA stability, alternative splicing, nucleosome modification, and noncoding RNA [27].

With the development of genetic technology, marker-assisted selection has been applied in many aquaculture species [28]. However, research developing microsatellite markers of disease resistance for fish is scarce. In this study, disease-resistance-associated microsatellite markers were developed from the Nile tilapia genome. To confirm that the microsatellite markers were associated with disease resistance, seven populations were chosen and analyzed by *Streptococcus* infection. As far as we know, this is the first report describing the development of disease-resistance-associated microsatellites in Taiwan tilapia. Thus, we hope our findings will have a positive practical impact on the tilapia industry in Taiwan.

## 2. Materials and Methods

### 2.1. Experimental Animals

**Figure 1:**
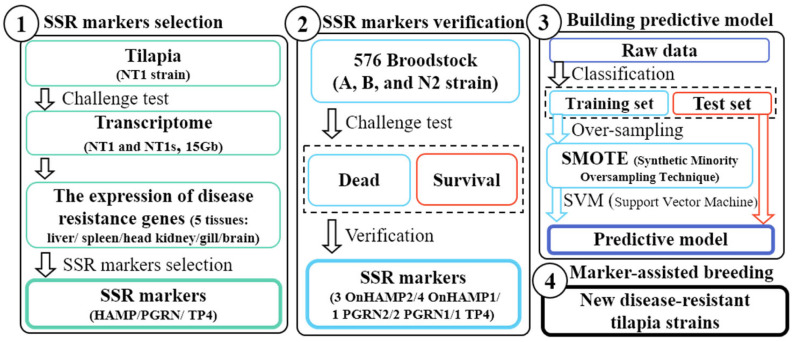
Experimental scheme showing the four main axes in this study. (**1**) SSR markers selection: transcriptome data were created by the NT1 strain challenge test, and SSR markers were selected through the transcriptome and gene expression results (NT1: NT1 strain without infection; NT1S: NT1 strain infected by 10^4^ CFU/g *Streptococcus iniae*). (**2**) SSR markers verification: a total of 576 fish from A, B, and N2 strains were infected with *S. iniae* to verify the association of SSR markers with disease resistance. (**3**) Building the predictive model: the raw data from step 2 were over-sampled by SMOTE method (synthetic minority oversampling technique), and then used to establish a predictive model through SVM (support vector machine). (**4**) Marker-assisted breeding: establish new disease-resistant tilapia strain by the marker-assisted breeding method.

Seven tilapia strains were used in this study. The NT1 strain is a Nile tilapia (*Oreochromis niloticus*) strain no.1 of National Taiwan Ocean University [29]. Two commercial Taiwan tilapia (*Oreochromis* spp.) strains, A and B strains, were collected from fish farms in south Taiwan. The Freshwater Aquaculture Research Center, Fisheries Research Institute provided the Nile tilapia (*Oreochromis niloticus*) N2 strain all-male XY fish obtained from crossing of YY supermale and XX female derived from N2 strain [30]. ANT1 (A male × NT1 female), AB (A male × B female) hybrid strains, and BB (B male × B female) strain are new *Streptococcus*-resistant strains. The transcriptome was generated through the NT1 strain and used for the selection of disease-resistance-related genes and SSR markers. SSR markers verification and the disease-resistant parental generation was performed by A, B, and N2 strains. Genetic inheritance of disease-resistant genotypes and confidence of predictive models were examined by ANT1, AB, and BB strains (Figure 1). All fish strains were cultured in a tank in 2T water at a stable temperature of 28 °C and were fed twice a day.

### 2.2. Transcriptome Generation

#### 2.2.1. Sample Collection

To generate tilapia transcriptome, the Nile tilapia NT1 strains (10 g, three months old) were prepared. NT1: NT1 strain without infection; NT1S: NT1 strain infected by *Streptococcus iniae* 89353 (10^4^ CFU/g body weight) [31] at 12 hpi. At 12 hpi by *S. iniae* 89353, five 3-month-old whole fish (10 g) from each group were sacrificed for total RNA extraction.

#### 2.2.2. Extraction of Total RNA

Approximately 0.1 *g* each of the liver, spleen, head kidney, gill, and brain tissues were individually collected in a 1.5 mL centrifuge tube. We then added 1 mL TRIzol and homogenized the mixture. The samples were incubated at room temperature for 5 min and centrifuged at 10,000× *g* for 2 min at 4 °C. Following this, we added 0.2 mL chloroform followed by vigorous shaking for 15 s and incubation at room temperature for 3 min. We then centrifuged the mixture at 10,000× *g* for 15 min at 4 °C. The supernatant liquid was transferred to a new centrifuge tube, 0.5 mL isopropanol was added, and the tube was mixed through gentle shaking. After incubation at room temperature for 10 min, the mixture was centrifuged at 10,000× *g* for 15 min at 4 °C, and the supernatant liquid was discarded. We then added 400 µL of 70% alcohol to the tube, following by gentle shaking and then centrifugation at 7500× *g* for 10 min at 4 °C. The supernatant was removed, and the sample was placed at 55 °C in an incubator for 3 min. Next, 50 µL DEPC-treated ddH_2_O (diethylpyrocarbonate-treated ddH_2_O) was added and the sample incubated at 55 °C for 15 min. A spectrophotometer was used to measure the ratio of OD_260_ to OD_280_ (with 1.9-2.0 corresponding to high purity). Finally, the samples were stored at −80 °C for later use.

#### 2.2.3. Purification of Total RNA

Using the PureLink RNA Mini Kit for purification of total RNA, we added 600 μL lysis buffer to the total RNA sample and vortexed until the mixture was homogenous. Then, we added 600 μL of 70% alcohol and vortexed evenly. A maximum of 600 μL of the mixture was transferred into a spin column, centrifuged at 12,000× *g* for 15 min at 4 °C, and this procedure repeated until the entire mixture was processed. Next, 350 μL Wash Buffer I was added, followed by centrifugation at 12,000× *g* for 25 s at 4 °C, and the filtrate was removed. Following this, 80 μL DNase I mixture (10 μL DNase and 70 μL RDD Buffer) was added followed by incubation at room temperature for 15 min. Then, another 350 μL Wash Buffer I was added, followed by centrifugation 12,000× *g* for 25 s at 4 °C, and the filtrate was removed. Next, 500 μL Wash Buffer II was centrifuged (at 12,000× *g* for 25 s at 4 °C, and the filtrate was removed by centrifuging at 12,000× *g* for 5 min at 4 °C; this procedure was repeated. The spin column was transferred into a recovery tube, and 20~50 μL of RNase-free water was then added, followed by incubation at room temperature for 2 min and centrifugation at 12,000× *g* for 2 min at 4 °C. RNA detection and quantification was conducted using a Nanodrop^TM^ 1000 spectrophotometer.

#### 2.2.4. Assembly, Function Annotation, and Differential Gene Expression

Trinity 12 software (r20140717, Broad Institute) [32] was used for de novo genome-guided assembly. The sequence used for reference was the published draft genome sequence of Nile tilapia/oreNil2 (oreNil2, Broad Institute of MIT and Harvard Orenil1.1 (GCA_000188235.1)). De novo transcriptome assembly of NT1 was submitted to the NCBI short read archive database (accession numbers: SRR14141863 and SRR14141864). 

The BAM file was generated from read mapping to the genome by TopHat (v2.0.13) [33] and the BAM file was assembled with Trinity 12. Subsequently, we mapped the reads to contigs using bowtie2 software (v2.2.3) [34]. The standardized count and FPKM of expression for all transcripts were calculated using RSEM (v1.2.0) [35]. We then calculated the expression and filtered the contigs so that the FPKM value was greater than 0.01. These contigs were compared with the Nile tilapia database (Oreochromis_niloticus.Orenil1.0.cdna.all.fa from http://ftp.ensembl.org/pub/release-78/fasta/oreochromis_niloticus/cdna/ accessed on 7 May 2015) through BLASTn (NT) (*p* < 0.00001).

The transcript DNA sequence was analyzed or compared using the vertebrate mammalian (VBMM: ftp.ncbi.nlm.nih.gov/refseq/release/vertebrate_mammalian/), the vertebrate non-mammalian (VBnonMM: http://ftp.ncbi.nlm.nih.gov/refseq/release/vertebrate_other/ accessed on 7 May 2015), SwissProt, KOG (EuKaryotic Orthologous Groups), KEGG (Kyoto Encyclopedia of Genes and Genomes), and GO (Gene Ontology) databases. The GO, KOG, and KEGG categories were annotated with BLASTX (with criteria: *e* value < 10^−5^) and the Blast2GO [36]. Finally, analysis of differential expression between any two samples was conducted by edgeR (V 3.10.3) [37].

### 2.3. The Gene Expression of Hamp in Tilapia

#### 2.3.1. Reverse Transcription

Total RNA (extracted and purified as described in Section 2.2.2 and Section 2.2.3) was reverse transcribed using a high-capacity cDNA reverse transcription kit. The sample was diluted to 100 ng/μL. We then mixed 10 μL RNA sample, 2 μL 10× RT Buffer, 0.8 μL 25× dNTP mix (100 mM), 2 μL 10X RT Random primer, 1 μL MultiScribe™ Reverse Transcriptase (50 U/μL), and 4.2 μL DEPC-treated ddH_2_O. PCR was conducted using a thermocycler (TProfessional Thermocycler, Biometra, BM-070-801), and the reaction conditions were the following: 1. 25 °C for 10 min; 2. 37 °C for 120 min; 3. 85 °C for 5 min. The sample was diluted 50X and stored at −20 °C for later use.

#### 2.3.2. Real-Time Quantitative PCR, qPCR

We used a Fast SYBR^®^ Green PCR Master for qPCR. The total volume was 20 µL and included 10 μL 2× Fast SYBR^®^ Green PCR Master Mix, 1 μL of 4 μM forward primer, 1 μL of 4 μM reverse primer, 3 μL DEPC-treated ddH2O, and 5 μL cDNA. The reaction included four steps: 1. 50 °C for 2 min; 2. 95 °C for 10 min; 3. 95 °C for 15 s and 60 °C for 60 s, repeated 40 times; 4. 95 °C for 15 s, 60 °C for 60 s, 95 °C for 15 s, and 60 °C for 15 s.

### 2.4. Selecting the Disease-Resistance-Associated Microsatellites

We searched for disease-resistance-associated microsatellites in the Nile tilapia genome (NCBI). Microsatellites can be classified on the basis of their size of the repeated sequence as follows: single nucleotide microsatellites ≥ 10 bp, dinucleotide microsatellites ≥ 6 bp, trinucleotide microsatellites ≥ 5 bp, tetranucleotide microsatellites ≥ 5 bp, pentanucleotide microsatellites ≥ 5 bp, and hexanucleotide microsatellites ≥ 5 bp [38]. We designed a microsatellite-specific primers using an online tool (Websat, http://wsmartins.net/websat/ accessed on 7 May 2015); primer length was 22 bp, Tm was 60 °C, GC was 60%, and the product length was between 100 and 400 bp [39].

### 2.5. Streptococcus iniae Challenge

#### 2.5.1. Collecting and Weighing Samples

Nile tilapia (body weight: 10–80 g) were used for the challenge experiment. The experimental group included F_0_ (NT1, A, B, and N2) and F_1_ (ANT1, AB, and BB) groups. To assess the effect of disease resistance, 20 fish were injected with PBS control at the same time. The fish were maintained in 30 L tanks at 28 °C following pathogen challenge. We continued the observation until no dead fish were observed for at least for three days. The fish were then used in the following experiments.

#### 2.5.2. *Streptococcus iniae* Culture

Liquid BHI was prepared for *Streptococcus iniae* 89353 cultures. A total of 30 μL *Streptococcus iniae* 89353 and 3 mL BHI broth were added into the autoclaved conical container. The mix liquid culture was left to grow overnight at 30 °C for 16 h. After 16 h, we cultured the entire aliquot of *S. iniae* in a larger volume for 8 h. This exact volume depended on the fish weight and the applied dose in subsequent procedures. The *S. iniae* 89353 bacteria were provided by the Bureau of Animal and Plant Health Inspection and Quarantine, Council of Agriculture, Executive Yuan, Kaohsiung Branch Dr. Benjia Zhao. Bacteria were re-isolated from a single symptomatic fish for biochemical confirmation of their identity.

#### 2.5.3. Intraperitoneal Injection

After anesthetizing the fish with 1 mL 2-phenoxyethanol (≥99%), the *S. iniae* was intraperitoneally injected into the tilapia with a lethal dose (LD) of 70–80 (strain A is 2×10^6^; B is 6×10^5^; N2 is 6.5×10^5^ CFU/g body weight). The control group was injected with PBS at the same volume of *S. iniae*. *S. iniae* was cultured in liquid BHI overnight (*Streptococcus iniae* culture as described in Section 2.5.2). Next, the dose was accurately calculated. The fish were monitored every day for the occurrence of death, and in this case, about 0.1 *g* of caudal fin was cut with sterilized scissors and placed into 70% alcohol for preservation. We continued observation until no dead fish were observed for at least three days. Finally, we cut approximately 0.1 *g* of fins from the surviving fish and stored samples in 70% alcohol.

### 2.6. Genomic DNA Extraction

Whole-genome DNA was extracted by a MasterPure^TM^ DNA Purification Kit. First, we removed the alcohol from the fin samples of tilapia after challenge and placed the sample into a new tube. We then added 300 μL tissue and cell lysis solution containing 50 μg of proteinase K, followed by thorough mixing and incubation of the solution at 55 °C for 16 h. Using a blunt pipette tip, we transferred the supernatant to a fresh microcentrifuge tube, added 175 μL of MPC protein precipitation regent, and mixed the solution gently, avoiding vortexing, as it may cause genomic DNA breakage. We centrifuged this mixture at 5000× *g* for 10 min at room temperature. We again used a blunt pipette tip to transfer the supernatant to a new microcentrifuge tube. Then, we added 500 μL of 100% isopropanol followed by thorough mixing. White filiform genomic DNA was visible during mixing, which was picked up using the tip and placed into a new microcentrifuge tube, and 500 μL of 70% ethanol was then added, followed by incubation at room temperature for 5 min. Ethanol was then removed, and the tube containing the DNA was placed in an incubator at 50 °C until the genomic DNA pellet turned transparent. Following this, 200~500 μL TE buffer was added and this mixture was incubated at 50 °C; gel electrophoresis was then used to confirm the quality of genomic DNA. The DNA concentration was measured using the nanodrop and the concentration of the solution was adjusted to 25 ng/μL before storage at −20 °C.

### 2.7. PCR Amplification of Fluorescently Labeled Microsatellite Products Using Specific Primers

The method to prepare fluorescently labeled microsatellite product was modified from standard multiplex PCR [40]. Primer details are shown in Table 1. First, we added 2 μL of 25 ng/μL DNA, 2.5 μL of 10× PCR-MgCl_2_ buffer, 0.5 μL of a 10 mM dNTP mixture, 0.75 μL of 50 mM MgCl_2_, 0.5 μL Platinum^®^ Taq polymerase, 0.5 μL of 10 μM forward primer, 0.5 μL of 10 μM reverse primer, and 17.75 μL ddH_2_O to the reaction tube (the total volume was 25 μL). The mixture was briefly centrifuged before PCR using a thermocycler (TProfessional Thermocycler, Biometra, BM-070-801). The reaction conditions were as follows: 1. 95 °C for 5 min; 2. 95 °C for 30 s; 3. 60 °C for 30 s; 4. 72 °C for 1 min; 5. 72 °C for 10 min. Next, steps 2–4 were repeated 35 times. This PCR reaction adds a G/C-rich adaptor sequence of 17 bases (5′GAGCACGAGGAGA3′) via inclusion in the 5′ site of all primers and serves as a site for binding site in the second PCR. Next, we took 2 μL of amplified product as a template and a fluorescently labeled primer was used as the forward primer for 5′ labeling of the product produced by the second polymerase chain reaction. The reaction time of the 3′ primer (reaction drug) was same as the first PCR reaction. The selection of products that were successfully labeled with the fluorescent primer was based on product size; products labeled with different fluorescent tags were similarly sized. The final product was stored at −20 °C.

### 2.8. Sequencing and Genotyping

We then checked the expected size of the product after microsatellite marker analysis. Next, the Digimage system (Major Science Digimage System DI-HD-110) was used to record images. To avoid excessive differences in fluorescence signals, we first used a Synergy^TM^ Muti-detection microplate reader to measure the concentration of each sample. Second, we quantified the concentration of each sample and adjusted samples to the same concentration. Third, we carried out equal volume mixing. The mixing was based on the same fluorescent marker of microsatellite product size, with differences of about 100–150 bp. Finally, we mixed the plural samples for the basic fluorescent peak and fluorescence signal analysis with an ABI 3730XL DNA analyzer. After the analysis, we used Genemaker software to analyze the microsatellite products size with different fluorescent markers and determined the genotype of individual fish.

### 2.9. Calculations and Statistical Analysis

#### 2.9.1. The Genetic Diversity Analysis

Genetic diversity analysis of tilapia populations with microsatellite markers was conducted using computer software GenAlEx 6.5 and the online tool Gene Calc (https://www.gene-calc.pl/pic). We included the number of alleles (N_a_), the effective number of alleles (N_e_), Shannon’s information index (I), allele frequency, genotype frequency, observed heterozygosity (H_o_), expected heterozygosity (H_e_), unbiased expected heterozygosity (uH_e_), polymorphic information content (PIC), individual fixation indices (F_IS_, F_IT_, and F_ST_), and the number of migrants per generation (N_m_) [41].

#### 2.9.2. The Correlation Analysis of Genotype and Survival Rate

The different genotypes were determined in the different groups, and the death or survival of fish after challenge was taken as a variable. Then, we performed Pearson’s chi-square test, correlation analysis, and the Z-test using IBM SPSS Statistics version 25.0 software (SPSS Inc., Chicago, IL, USA). Moreover, post hoc Bonferroni correction was used to adjust the *p* value. α0*k* = α0′ was mentioned in the Bonferroni correction, where k represents the total examination number. 

### 2.10. The Effectiveness Analysis of Genotypes by Predictive Models 

This technique uses a support vector machine [42] to build the predictive model with an IBM SPSS modeler subscription (version SaaS). The process of building and applying a predictive model has three basic steps: 1. Building a predictive model; 2. Testing the predictive model; 3. Applying the predictive model.

#### 2.10.1. Building a Predictive Model

The predictive model established in this study predicts the number of death events following *S. iniae* challenge, and also predicts the mortality rate. A total of 384 fish from the A and B strains were selected to train the model. Eight predictors were chosen, including SSR2, SSR4, SSR7, SSR14, SSR18, SSR19, SSR21, and SSR22. The event of death was chosen as the target (survival: 0; death: 1). In total, 90% of all samples were randomly selected as the training set and 10% were chosen as the testing set. To balance the efficacy of both the dead and alive groups, the synthetic minority oversampling technique [43] was used as an imbalanced data processing method. The testing set was an independent set and was not used for SMOTE and training purposes. 

#### 2.10.2. Evaluation of the Predictive Model

First, we made a confusion matrix containing a true positive (TP), a true negative (TN), a false positive (FP), and a false negative (FN). These four parameters were then used to calculate the true positive rate (TPR), the true negative rate (TNR), the positive predictive value (PPV), the negative predictive value (NPV), the false positive rate (FPR), the false discovery rate (FDR), the false negative rate (FNR), accuracy (Acc), the F1 score, the Matthews correlation coefficient (MCC), the receiver operating characteristic curve (ROC), and the area under curve (AUC) for the evaluation model [44,45,46].


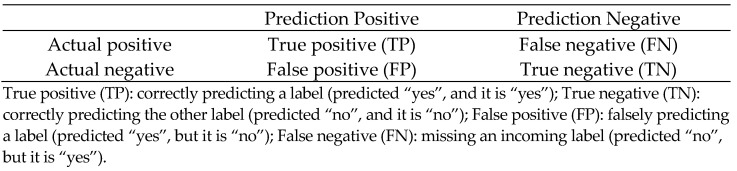


True positive rate (TPR): additionally known as sensitivity (Sn) recall, this is the proportion of samples that are judged to be positive of those that are actually positive and is calculated using the following equation:

(1) TP/(TP+FN)

True negative rate (TNR): additionally known as specificity (Sp), this is the percentage of samples judged to be negative of those that are actually negative and is calculated using the following equation:

(2) TN/(TN+FP)

Positive predictive value (PPV): additionally known as precision, this is a measure of what percentage of the sample that is predicted to be positive is actually positive, and the equation is as follows:

(3) TP/(TP+FP)

Negative predictive value (NPV): the definition is the same meaning as for PPV, except negative (negative predictive value) instead; the equation is as follows:

(4) TN/(TN+FN)

False positive rate (FPR): the number of actually negative samples that were predicted as positive as a ratio of all the actually negative samples; the equation is as follows:

(5) FP/(FP+TN)

False discovery rate (FDR): the proportion of samples predicted to be positive that are actually negative; the equation is as follows:

(6) FP/(TP+FN)

False negative rate (FNR): additionally known as the miss rate, this is the number of samples predicted to be negative that are actually positive as a ratio of true positive samples; the equation is as follows:

(7) FN/(FN+TN)

Accuracy (Acc): the ratio of the sample correctly classified by the classifier to the total number of samples for a given test dataset, i.e., the probability of correct prediction; the equation is as follows:

(8) (TP+TN)/(TP+TN+FP+FN)

*F*_1_ score: the equation to measure the balance between true positive rate (TPR) and positive predictive value (PPV) is as follows:

(9)   2TP/(2TP+FP+FN)

Matthews correlation coefficient (MCC): MCC is essentially a correlation coefficient describing the correlation between the actual and predicted classifications and has values in the range [−1, 1]. A value of 1 indicates perfect prediction of the subject, a value of 0 indicates that the prediction is not as good as random prediction, and −1 means that the predicted classification and the actual classification do not agree at all [46]. The equation is as follows:

(10) (TP×TN−FP×FN)/√((TP+FP)×(TP+FN)×(TN+FP)×(TN+FN) )

Receiver operating characteristic curve (ROC curve): the ROC curve is a two-dimensional coordinate system where the false positive rate (FPR) is the x-axis and the true positive rate (TPR) is the y-axis.

Area under curve (AUC): in addition to the shape of the curve, the area under curve (AUC) can also be used to determine the discriminatory power of the ROC curve. The AUC value ranges from 0 to 1; the larger the value, the better. The following are the general rules for determining the AUC value [47,48]:

AUC = 0.5 (no discrimination), ROC is just diagonal.

0.7 < AUC < 0.8 (acceptable discrimination).

0.8 < AUC < 0.9 (good discrimination).

0.9 < AUC < 1.0 (excellent discrimination).

The equation is as follows, where t is FPR and ROC (t) is TPR:

(11) AUC=∫_0^1▒ROC(t)dt

#### 2.10.3. Applying a Predictive Model

To predict the disease resistance of new strains with a predictive model, three strains were selected (ANT1, AB, and BB strains). The SVM results are described as follows:

$S-fieldname: the predicted value of target field. Here, the target is death (1). 

$SP-fieldname: probability of predicted value.

$SP-value: probability of each possible value of the flag, alive (0) or dead (1).

### 2.11. Detecting the Disease Resistance of New Strains

Nile tilapia (body weight: 10-80 g) were used for the challenge experiment. The experimental groups are new F_1_ strains (ANT1, AB, and BB strains). To assess the effect of disease resistance, 20 fish were injected with PBS control at the same time. The fish were maintained in 30 L tanks at 28 °C after challenge. We continued observation until there were no dead fish at least for three days.

## 3. Results

### 3.1. Transcriptome Analysis 

More than 50 million raw reads were assembled into 129,105 contigs (mean size: 1086.21 bp; range: 201–38,020 bp). The transcript DNA sequences were analyzed using KOG (EuKaryotic Orthologous Groups), KEGG (Kyoto Encyclopedia of Genes and Genomes), and GO ontology databases. Table S1 shows that 59,393 transcripts are annotated in KOG. A total of 11,429 transcript IDs were assigned to 377 KEGG pathways. According to the *p* value, Table S2 presents the top 50 KEGG pathways. In the GO annotation, 22,690 transcript IDs were annotated to 56 categories (Table S3). According to this result, seven differentially expressed transcripts related to disease resistance were found, including four hepcidin-*,* two progranulin-*,* and one piscidin-related transcripts (Table S4).

### 3.2. The Gene Amplification of Hamp

**Figure 2:**
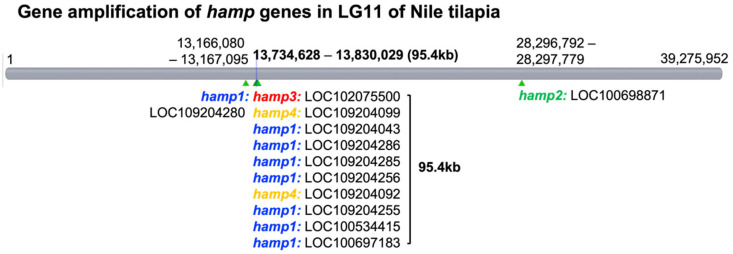
The gene amplification of hepcidin *hamp* genes in LG11 of Nile tilapia. There are 12 *hamp* genes including 8 *hamp1*, 1 *hamp2*, 1 *hamp3,* and 2 *hamp4* genes distributed into three locations indicated by green arrow heads in the LG11 of Nile tilapia.

**Figure 3:**
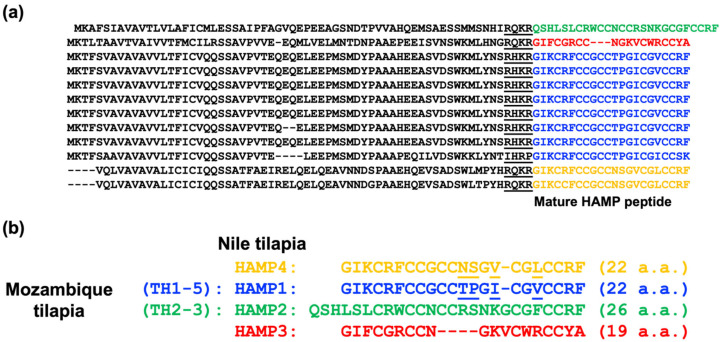
Twelve Nile tilapia *hamp* genes in LG11 encode four HAMPs: (**a**) the mature HAMP peptide sequences in Nile tilapia. (**b**) Comparison of the HAMP mature peptides of Mozambique tilapia and Nile tilapia. The HAMP mature peptides Th1-5 and Th2-3 of Mozambique tilapia are identical to HAMP1 and HAMP2 of Nile tilapia, respectively. Moreover, HAMP3 and HAMP4 are only found in Nile tilapia.

The gene amplification of *hepcidin*/*hamp* was found in the LG11 of the Nile tilapia whole-genome database by Genome Data Viewer in NCBI website, forming 12 genes, including 8 *hamp1*, 2 *hamp4*, 1 *hamp2*, and 1 *hamp3* genes. The *hamp* genes were distributed into three locations including one gene cluster containing 10 *hamp* genes (7 *hamp1*, 1 *hamp3,* and 2 *hamp4*) spanning 95.4 kb, and one individual *hamp1* gene and one *hamp2* gene in LG11 (Figure 2). These 12 *hamp* genes in LG11 encode 4 HAMPs (Figure 3).

### 3.3. The Expression of Hamp Genes Associated with Disease Resistance 

**Figure 4:**
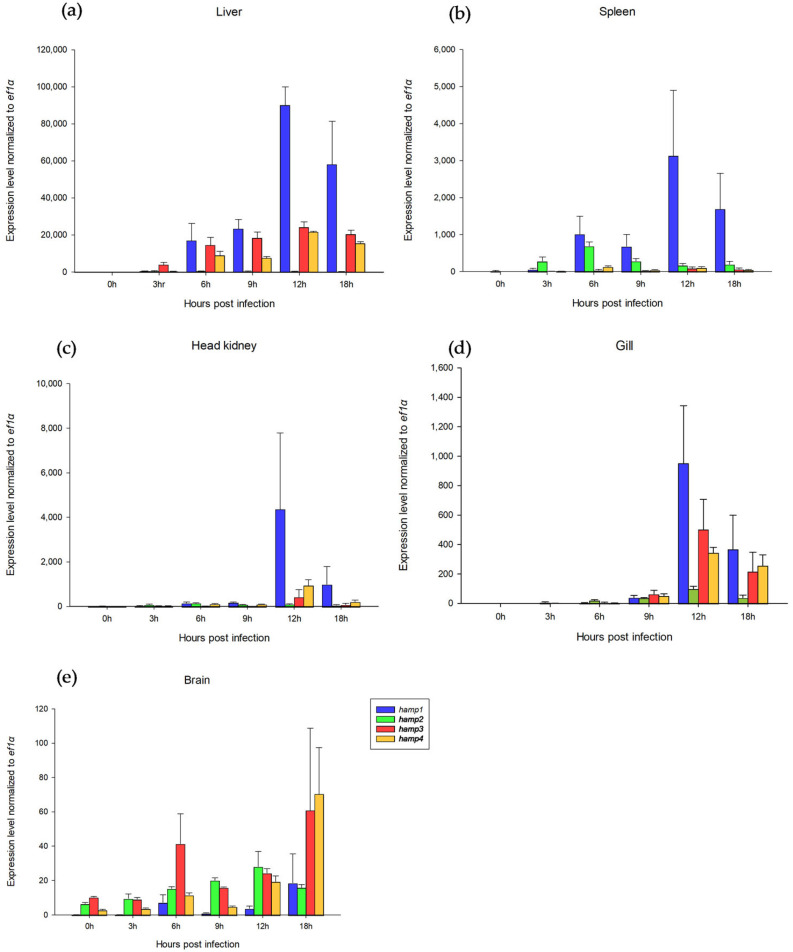
The gene expression of *hamp1*, *hamp2*, *hamp3*, and *hamp4* in the NT1 Nile tilapia during *Streptococcus iniae* infection (1.4×10^5^ CFU/g) by qPCR. Liver, spleen, head kidney, gill, and brain tissues were collected at 0, 3, 6, 9, 12, and 18 h after infection. The gene expression was calculated by 2^−ΔΔC^_T_, and *ef1α* was the reference gene. Each bar is the mean + SEM of three independent fish: (**a**) the gene expression of *hamp1*, *hamp2*, *hamp3*, and *hamp4* in the liver of tilapia during *S. iniae* infection; (**b**) spleen; (**c**) head kidney; (**d**) gill; (**e**) brain.

NT1 Nile tilapia (body weight: 100~400 g) were selected. *S. iniae* was intraperitoneally injected into the tilapia with a lethal dose of 50 (1.4 × 10^5^ CFU/g). Liver, spleen, head kidney, gill, and brain tissues were collected at 0, 3, 6, 9, 12, and 18 h after infection for qPCR detection. In the liver, *hamp1* levels increased from 6 h and reached a maximum at 12 h, which was 46,075 times the level at 0 h (uninfected); *hamp2* levels reached a maximum at 3 h, 14.9 times higher than those at 0 h; *hamp3* levels increased from 3 h and reached a maximum at 12 h, 2741 times higher than at 0 h; *hamp4* levels increased from 6 h and reached a maximum at 12 h, 46,312 times the height of 0 h. The genes in descending order of expression were *hamp1*, *hamp3*, *hamp4*, and *hamp2* (Figure 4a). In the spleen, *hamp1* and *hamp3* levels increased from 6 h and reached a maximum at 12 h; *hamp2* levels increased from 6 h and reached a maximum at 12 h, 37 times higher than at 0 h; *hamp4* levels reached a maximum at 12 h, 290 times higher than levels at 0 h. The genes in descending order of expression were *hamp1*, *hamp2*, *hamp4*, and *hamp3* (Figure 4b). In the head kidney, *hamp1, hamp3*, and *hamp4* levels reached a maximum at 12 h; *hamp2* levels increased from 3 h and reached a maximum at 6 h, 14 times higher than levels at 0 h. The genes in descending order of expression were *hamp1*, *hamp4*, *hamp3*, and *hamp2* (Figure 4c). In the gill, all the *hamp* genes’ expression levels reached a maximum at 12 h. The genes in descending order of expression were *hamp1*, *hamp3*, *hamp4*, and *hamp2* (Figure 4d). In the brain, *hamp1, hamp3*, and *hamp4* levels increased from 6 h and reached a maximum at 18 h; *hamp2* levels increased from 3 h and reached a maximum at 12 h, 6 times higher than the levels at 0 h. The genes in descending order of expression were *hamp4*, *hamp3*, *hamp2*, and *hamp1* (Figure 4e).

### 3.4. Disease-Resistance-Associated Microsatellites

In total, we found 17 microsatellites linked to the *hamp* gene family in LG11, 4 microsatellites linked to the short type *pgrn* family in LG22, and 1 microsatellite linked to the tilapia piscidin family in LG15 (Table 1). Table S5 shows details of the specific primers that were used.

### 3.5. Selection of Disease-Resistant Tilapia by Streptococcus iniae Challenge

Before selection of disease-resistant tilapia, 20 fish from the A, B, and N2 strains were infected by *S. iniae* to understand the LD (lethal dose) of each tilapia group. The results revealed that the LD70-80 of strain A was 2 × 10^6^ CFU/g (colony forming unit per gram of body weight), strain B was 6×10^5^ CFU/g, and strain N2 was 6.5 × 10^5^ CFU/g in tilapia following *S. iniae* challenge. After LD test of each group, 200 (strain A), 198 (strain B), and 197 (strain N2) fish were challenged by *S. iniae* (6.5 × 10^5^ to 2 × 10^6^ CFU/g). As a result, 144 fish died in strain A, 145 fish died in strain B, and 157 fish died in strain N2 after 14 days (Table S6). In all the strains, the highest mortality was found on the first and second days, and the survival rate was stable on the last three days of the study (12–14).

### 3.6. The Genotype Analysis of the Disease-Resistance-Associated Microsatellites in Tilapia

The results based on the examination of 17 SSRs linked to *hamp* family genes, 4 SSRs linked to *progranulin* family genes, and 1 SSR linked to *tilapia piscidin* family gene as microsatellites associated with disease resistance in tilapia are shown in Table 1. After detection, seven *hepcidin*, three *progranulin*, and one *tilapia piscidin* family gene associated with microsatellites generated the specific product with fluorescence, including three OnHAMP2: SSR2 SSR4, SSR5; four OnHAMP1: SSR7, SSR8, SSR14, SSR17; one PGRN2: SSR18; two PGRN1: SSR20, SSR21; and one TP4: SSR22. The genotype frequency and alleles frequency of microsatellite markers were counted by GenAlEx 6.5 software (genetic analysis in Excel), and 106 alleles and 271 genotypes were found. The results of allele frequency are depicted in Table S7.

### 3.7. The Genetic Diversity Analysis of the Disease-Resistance-Associated Microsatellites in Tilapia

Ten disease-resistance-associated microsatellites in the A, B, and N2 strains of tilapia were detected using the GenAlEx 6.5 software, and 62, 70, 46 alleles were identified, respectively. The mean N_a_ was 6.2 ± 0.512, and no fixed (monomorphic) locus was found. Mean N_e_ was 3.015 ± 0.314, lower than mean N_a_; mean I was 1.247 ± 0.104; mean H_o_ was 0.634 ± 0.051; mean H_e_ was 0.633 ± 0.039; mean H_o_ was slightly higher than mean H_e_. Mean *u*H_e_ was 0.635 ± 0.039; mean F_IS_ was −0.042 ± 0.116; mean PIC was 0.583 ± 0.163. Afterward, in the B population, mean N_a_ was 7 ± 1.135, and no fixed (monomorphic) locus was found. Mean N_e_ was 3.907 ± 0.368, lower than mean *N_a_*. Mean I was 1.485 ± 0.126; mean H_o_ was 0.591 ± 0.06; mean H_e_ was 0.718 ± 0.033; mean *H_o_* was lower than mean H_e_. Mean uH_e_ was 0.72 ± 0.033; mean F_IS_ is 0.167 ± 0.084; mean PIC was 0.575 ± 0.122. In strain N2 tilapia, mean *N_a_* was 4.6 ± 0.653, and no fixed (monomorphic) locus was found. Mean *N_e_* is 2.786 ± 0.345, lower than mean *N_a_*. Mean *I* was 1.079 ± 0.125; mean *H_o_* was 0.656 ± 0.095; mean *H_e_* was 0.591 ± 0.048; mean H_o_ was higher than mean H_e_. Mean uH_e_ was 0.592 ± 0.048; mean *F_IS_* was −0.057 ± 0.109; mean PIC was 0.527 ± 0.170. The results of the genetic diversity analysis are presented in Table S8. Then, three groups were compared in pairs. The results revealed that the *F_ST_* of the A population to the B population was 0.114, and the Nm was 1.950; the F_ST_ of the A population to the N2 population was 0.102, and the Nm was 2.191; the F_ST_ of the B population to the N2 population was 0.098, and the Nm was 2.294. From highest to lowest, number of migrants per generation, the order of strains was N2, B, and A (Table S9).

### 3.8. The Correlation Analysis of Genotype and Survival Rate of the Disease-Resistance-Associated Microsatellites in Tilapia

Table 2 presents 10 of the 11 microsatellite markers that were found to have a statistically significant association (*p* < 0.05) after the correlation analysis between genotype and survival by chi-square test. The significant markers were SSR2, SSR4, SSR5, SSR8, SSR14, SSR17, SSR18, SSR19, SSR21, and SSR22. Furthermore, all correlations were at a highly significant level (*p* < 0.001), except for SSR5. However, only 1 of the 11 microsatellite markers (SSR5) was statistically significant (*p* < 0.05) after the correlation analysis by the chi-square test in strain B (Table 3). Table 4 presents 3 of the 11 statistically significant microsatellite markers (*p* < 0.05) after the chi-square test in the N2 strain, including SSR2, SSR14, and SSR22. Moreover, the correlations of SSR22 were at a highly significant level (*p* < 0.001). Furthermore, the associations between all genotypes of each SSR and the number in the alive or dead groups were analyzed by Z-test. There were four microsatellite markers (SSR4, 5, 7, 19) in the B strain and five microsatellite markers (SSR2, 14, 17, 19, 22) in the N2 strain that showed a statistically significant difference (*p* < 0.05) (Table S10).

### 3.9. Predictive Model and the Effectiveness Analysis of Genotypes 

For understanding the effectiveness and heritability of genotypes, predictive modeling was established for 384 fish from strains A and B (F_0_). In the process of model establishment, 90% of samples were used for training and 10% of samples were used for testing, and death was used as the target (survival: 0; death: 1). Eight SSR markers were chosen for predictors (SSR2, SSR4, SSR7, SSR14, SSR18, SSR19, SSR21, and SSR22). To avoid imbalance and overfitting of data, the synthetic minority oversampling technique (SMOTE) was used as the processing method for data balancing. The SMOTE results are shown in attachment 1 (including raw training set (90%) and independent testing set (10%); adjective training set via SMOTE; predictive results). 

Table 5 presents the confusion matrix of the training and testing sets. In the training set, true positive (TP) is 242, false positive (FP) is 65, true negative (TN) is 181, and false negative (FN) is 4. In the testing set, TP is 26, FP is 5, TN is 7, and FN is 1. Then, the evaluation values of the predictive model were calculated through the four parameters of TP, FP, TN, and FN (see Table 6). Figure S1 shows the receiver operating characteristic curve (ROC curve) with FPR (1-specificity) and TPR (sensitivity). Meanwhile, we calculated the area under curve (AUG). The AUG was 0.983 in the training set and 0.849 in the testing set.

**Figure 5:**
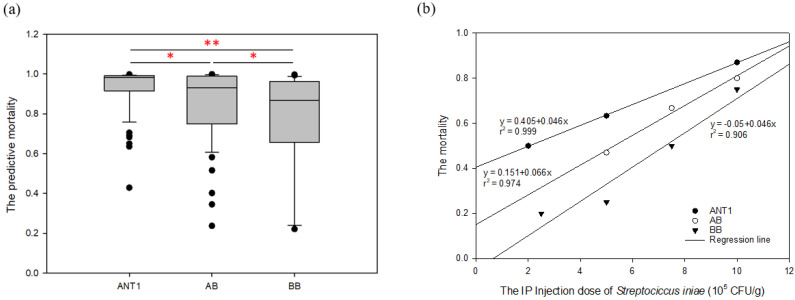
The predictive and actual mortality of new *Streptococcus*-resistant groups (F_1_): (**a**) the predictive mortality of tilapia groups via the SVM predictive model. The x-axis denotes different groups, i.e., 96 fish in ANT1; 55 fish in AB; and 40 fish in the BB group. The y-axis is the predictive mortality via the SVM predictive model; 1.0 denotes 100% mortality. The gray box represents the interquartile range (IQR); from top to bottom are upper quartile (Q3), median (Q2), and lower quartile (Q1), respectively. The upper line is maximum and the lower line is minimum; dark points denote an outlier. *: there is a statistically significant difference by one-way ANOVA and Tukey post hoc test (*p* < 0.05); **: *p* < 0.01. (**b**) The mortality of tilapia groups with regression lines through *S. iniae* IP injection. The x-axis is the IP injection dose of *S. iniae* (10^5^ CFU/g). The y-axis is the mortality after *S. iniae* IP injection; 1.0 signifies 100% mortality.

To determine the reliability of the predictive model, the mortality of the new *Streptococcus*-resistant group (F_1_) was calculated using the predictive model. In total, 96, 55, and 40 fish were selected from strains ANT1, AB, and BB, respectively. The experimental results exhibited the predicted value and probability of predicted value (predicted probability). The results show that death was predicted for 95, 52, and 31 samples in groups ANT1, AB, and BB, respectively (Table S11). Figure 5a shows the predictive mortality of tilapia groups using the predictive model. The mean predictive mortality (±SEM) of ANT1 is 0.932 ± 0.0107, AB is 0.861 ± 0.0245, and BB is 0.765 ± 0.0414. The differences between the ANT1 and the AB, and BB groups are significant. The new hybrid strains (F_1_) were infected by *S. iniae* to further establish the prediction accuracy of the predictive models. Figure 5b presents the mortality and the IP injection dose of *S. iniae* (2–10 × 10^5^ CFU/g) with regression lines.

## 4. Discussion

At the beginning of this study, the NT1 strain was infected by *S. iniae* 89353 (10^4^ cfu/g) at 12 hpi for the collection of the transcriptome sample. In contrast to previous research [49,50], a lower dose and longer response time were selected to allow observation of the recovery response after challenging with *S. iniae*. Even if there was no enormous differential gene expression in the transcriptome result (Table S4), the qPCR results indicate that *hamp* gene expression increased substantially. Figure 4 shows the *hamp1*, *hamp3*, and *hamp4* levels reaching a maximum at 12 h (*hamp2* at 3–6 h) in all tissues during *S. iniae* infection. It was also reported that hepcidin expression increased remarkably after pathogenic infection [51,52]. Figure 2 and Figure 3 present the results for 12 hepcidin genes in Nile tilapia. These results are similar to previous reports showing that multiple hepcidin gene copies have been generated through duplication and diversification processes in fish [19,53,54,55], and that Nile tilapia has more hepcidin genes than blue tilapia [18]. These results suggest that hepcidin gene amplification is associated with disease resistance in tilapia. These genes were found to be significantly upregulated after the challenge experiment. Surprisingly, hepcidin gene amplification was also observed (Figure 2 and Figure 3). It is therefore assumed that these genes play an important role in the infective response. Furthermore, the short product of *pgrn* could enhance disease resistance via participation in the regulation of innate immune-related genes in tilapia [56]. The GRN-41 peptide, which is product of *Pgrn1* generated by alternative RNA splicing, also has antimicrobial activity against *Vibrio* [57]. The disease resistance of tilapia can be effectively increased by TP4 (tilapia piscidin 4) [58,59,60,61].

The correlation analysis by chi-square test (Table 2, Table 3 and Table 4) and Z-test (Table S10) suggest the relationship between SSRs polymorphism and disease resistance. Moreover, Figure 4 indicates the association of gene expression and disease resistance in HAMP. We hypothesized that different SSRs lengths affect gene expression, which in turn caused effects on disease resistance. The effect of SSRs on gene expression has been reported [26,27]. Some of the examples of SSRs affecting gene expression are as follows. In human, there was a long CGG trinucleotide repeat in the 5′-UTR of the *FMR1* gene. This SSR was adjacent to the promoter and affected the performance of the *FMR1* gene, leading to fragile X syndrome (FXS) [62]. In another study, the (GA)n microsatellite sequence of the promoter region has been shown to bind the GAGA factors (GAF) proteins in *Drosophila* GAGA factor (GAF) [63,64]. The GAF is a multifunctional protein that influences gene expression, the communication between promoters and enhancers, nucleosome organization, and chromosome structure [65]. In mammalian cells, the transcription start site (TSS) at the 5′-UTR end of the promoter is affected by GAF binding sequences [66]. Streelman and Kocher present the (CA)n microsatellite which is found in the prolactin 1 (*prl 1*) gene of the 5′-UTR is associated *prl 1* gene expression in tilapia [67]. Both *prl1* and growth hormone (GH) gene polymorphism has been proven to be linked to growth in tilapia [68]. Overall, our study is based on these disease-resistance-related genes of tilapia, and we selected samples for intraperitoneal injection of the highly pathogenic *S. iniae*. Developing microsatellites associated with these antimicrobial peptides might effectively aid in the marker-assisted selection of disease-resistant strains of aquaculture species. 

In the above-mentioned study, we concluded that SSR and disease resistance are indeed associated, but the results of these markers are not similar to different strains. As the result, the correlation analyses of A, B, and N2 populations were, respectively, 10, 1, and 3 of the 11 microsatellite markers after the correlation analysis between genotype and survival by the chi-square test (*p* < 0.05). Then, there were four microsatellite markers (SSR4, 5, 7, 19) in the B strain and five (SSR2, 14, 17, 19, 22) in the N2 strain (Table S10), both of which showed a statistically significant difference (*p* < 0.05) when analyzing the association between all genotypes of each SSR and the numbers in the alive or dead groups by Z-test. These results indicate that the disease resistance of strain A is higher than in strains B and N2, and B and N2 strains are similar. The direct reason may be the variation in disease resistance of different strains. This result is also consistent with the result of the *S. iniae* challenge experiment (the LD71.3 of strain A was 2 × 10^6^ CFU/g, the LD73.2 of strain B was 6 × 10^5^ CFU/g, and the LD79.7 of strain N2 was 6.5 × 10^5^ CFU/g).

**Figure 6:**
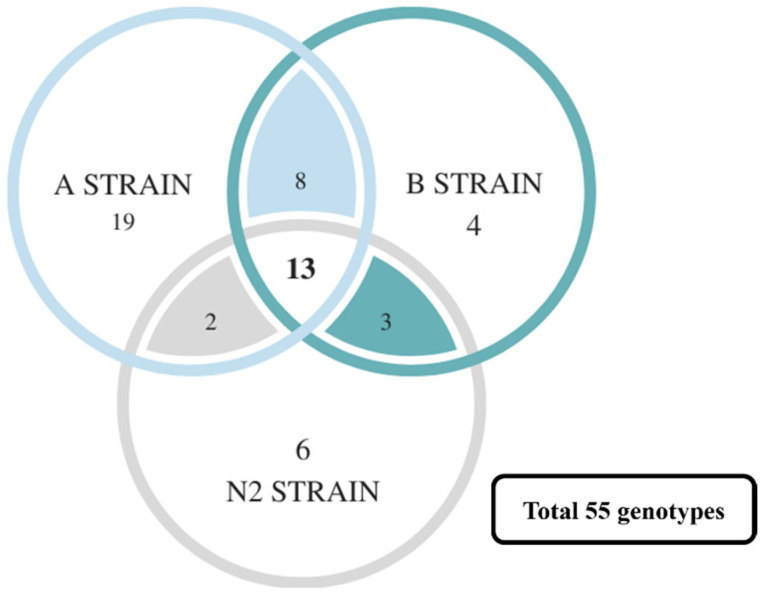
The Venn diagram of disease-resistance-associated genotypes in A, B, and N2 strains. Total number of genotypes is 55.

Furthermore, the correlation analysis results of all SSRs in the A, B, and N2 strains were compared. A total of 271 genotypes were found, of which 55 genotypes were related to survival (Table S10). However, many survival-related genotypes were only found in specific strains (such as SSR5, 8, and 21), even having a statistically significant difference (especially strain A, Figure 6). This may because of the following: (1) the number of genotypes is too large, but the number of fish related to each genotype is too small; or (2) the challenge experiments with a high lethal dose create a smaller number of survivor groups. Nevertheless, the SSR5, SSR8, SSR19, and SSR22 in strain A are still significantly related to survival. It was found that the number and proportion of most genotypes that have no significant difference in other groups were still associated with survival via a comparison with the number and proportion of all microsatellite markers in the death and survival groups (Table S10). Fuji et al. [69] mentioned that, despite there being no significant correlation in the first generation of statistics, the ratio of certain genotypes could be increased by repeated backcrossing; thus, a microsatellite closely linked to lymphocystis disease resistance (LD-R) was selected from 50 microsatellites. They also attempted to transfer the closely linked LD-R microsatellite marker Poli9-8TUF into a commercial strain, and they successfully developed a new disease-resistant strain [70]. These findings were corroborated in our study; although some genotypes are only significantly associated with survival in specific strains, they are nevertheless associated with survival.

Although 55 disease-related genotypes were found by a Z-test, most genotypes were found in strain A. It was proposed that more disease-resistant markers could be found in the strain demonstrating broader disease resistance. However, those with both significant differences and no significant differences might still be potential molecular markers. As mentioned in the previous section, even if 37 genotypes only have a significant difference in strain A, the number and proportion of most genotypes which have no significant difference in other strains are still associated with survival. Moreover, disease resistance may also be graded according to different combinations of genotypes. Our hypotheses are the following: (1) every genotype has different strengths; and (2) different combinations of genotypes cause variations in effectiveness. To address the challenges arising from this variation, predictive modeling was built by strains A and B.

Table 6 shows the evaluation values of our predictive model. Accuracy is the factor most commonly evaluated for a predictive model and is represented by a value derived from the number of samples of correct judgment (true positive and true negative) divided by all samples [71]. Accuracy was 0.8598 in the training set and 0.8462 in the testing set (range from 0 to 1; the closer to 1, the better). However, this accuracy does not apply when the actual number of positive samples is low; thus, precision and sensitivity are used. Precision and sensitivity are both concerned with a true positive, but from different perspectives. Precision relies on predicting the actual precision in a positive situation, while sensitivity predicts “how much” of the actual positive answer can be recalled in a positive situation [45]. The precision in the training set was 0.7883 and the sensitivity was 0.9837. The precision in the testing set was 0.8387 and the sensitivity was 0.9630. Both values were close to 1 (especially the sensitivity). The *F_1_*-score is the harmonic average of the two values (precision and sensitivity) and is often used to evaluate the accuracy of a given model [45]. The *F_1_*-scores were 0.8752 and 0.8966 for the training and test sets, respectively (range from 0 to 1; the closer to 1, the better). The sensitivity denotes detection of how many samples will actually die. The specificity represents the number of samples that survived via predictive model detection. The higher the sensitivity and specificity values, the better the model in terms of prediction (range from 0 to 1; the closer to 1, the better) [72]. The specificity for the training set and the test set was 0.7358 and 0.5833, respectively, which indicated that the actual surviving samples in the test set were slightly lower than in the training set. MCC is usually regarded as a balanced indicator. In essence, MMC is a correlation coefficient that describes the actual classification and the predicted classification. The range is from −1 to 1. A value of 1 describes a perfect prediction, a value of 0 shows that the predicted result is worse than the random result, and −1 demonstrates that the predicted classification and the actual classification are completely inconsistent [46]. The MCC in the training set and testing set was 0.7427 and 0.6244, respectively. Additionally, the closer the FPR, FDR, and FNR are to 0, the better. The results showed that only the FPR of the testing set was higher. This is because FPR = 1 − specificity, and a lower correct number of tested actual surviving samples means a higher incorrect number of tested surviving samples. Therefore, we also plotted the ROC curve (Figure S1) using false positive rate (FPR) and true positive rate (TPR). If the ROC curve is equal to the diagonal line, the model shows no discrimination. If the ROC curve moves to the upper left corner, the model is more sensitive to disease resistance (the lower false positive rate), which means the model has better discrimination. Meanwhile, the ROC curve is used to calculate the area under curve, which ranges from 0 to 1; the larger value, the better [44,45,47]. The AUC was 0.983 in the training set, which meant excellent discrimination. The AUC was 0.849 in the testing set, which meant good discrimination. Combining the evaluation values from Table 6 and Figure S1, it is proposed that this model has certain credibility in predicting the mortality of a population.

The model was applied to detect the new *Streptococcus*-resistant group (F_1_). The result reveals that the proportions of predictive death numbers in the ANT1, AB, and BB groups were 95/96 (0.99), 52/55 (0.945), and 31/40 (0.775), respectively. Meanwhile, the mean predictive mortality (±SEM) of ANT1 was 0.932 ± 0.011, AB was 0.861 ± 0.026, and BB was 0.765 ± 0.041 (Table S10). Moreover, there are more samples with lower mortality in the BB group (Figure 5a). As a result, the trend of predictive disease resistance (Figure 5a) corresponded with actual disease resistance (Figure 5b). The mortality of ANT1 was highest, next was AB; BB was the lowest group. The predictive model, which was built by disease-resistance-associated microsatellites, could not only predict the mortality of a pure line but also of hybrid offspring. These results may indicate that the resistance-related genotypes which are found from F_0_ are still applicable in the offspring. Moreover, this predictive model can also estimate the mortality rate in genotype combinations. There are still numerous issues with this model: (1) the *S. iniae* dose does not ensure mortality and only an approximate relative value; (2) offspring not sharing the same genotype as their parent leads to inaccurate interpretation; and (3) the sample size may be too small to build a predictive model. Incorporating more information will increase the accuracy of predictions. To establish a precise predictive model, the dose of the challenge experiment and the time of death should be added to this SVM model, or different models should be established in further experiments. In addition, the predictive model could also be strengthened via machine learning during the breeding process.

With the development of molecular biotechnology, a huge variety of genome-based biotechnologies have been applied to the field of aquaculture research. However, most Nile tilapia breeding relies on traditional breeding methods to select phenotypes, such as growth rate, weight, and length. Relative to the aquaculture industry, modern genome-based strategies (e.g., marker-assisted selection and genomic selection breeding) have been widely using in agriculture and animal industries. Even though marker-assisted selection has only begun to be applied in the aquaculture industry in recent years, some cases of aquaculture studies can be found; for instance, high growth rate, cold resistance, and disease resistance in flatfish [69,70], rainbow trout [73,74,75,76,77], and carp [78,79]. Thus far, most of the current research of marker-assisted selection has focused on developing massive SNPs, SSRs, and deletions in Nile tilapia [80] for sex determination [81], population structure analysis [82], improvement of growth and fillet yield [83,84], and cold stress [85]. Often, few markers are used in breeding, which is not only time consuming but expensive. Overall, there are still only a few areas of research into disease-resistance-associated microsatellites in tilapia, but all commercial tilapia strains in Taiwan are hybridized. Previous studies may therefore not provide precise information directly relevant for the Taiwan tilapia industry.

## 5. Conclusions

In this study, 11 disease-resistance-associated microsatellites and 55 survival-related genotypes were identified and characterized and a predictive model for mortality linked to disease was developed. Three new *Streptococcus*-resistant strains were established through a double challenge experiment and marker-assisted selection. Even though some studies have reported that researchers successfully established *Streptococcus*-resistant tilapia, it is necessary to establish many families at the same time. Moreover, disease resistance in previous research could only be detected through challenge experiments or exposure to environmental stresses. This will be tough to use in the farms. Our findings allow selection of disease-resistant fish without gene expression. It may provide a cost-effective and time-saving strategy for assessing disease resistance and can accelerate the breeding process with the use of fewer fish, families, offspring, and markers and without the need for killing.

## Figures and Tables

**Table 1:** Disease-resistance-associated microsatellites. Total of 22 SSRs, including 17 linked to the *hamp* gene family, 4 linked to the short type *pgrn* family, and 1 linked to the tilapia *piscidin* family.

SSR	Associated Gene	SSR Name	SSR
SSR1	HAMP2 in LG11 (ID: 100698871) 5′end 3.8 kb	OnHAMP2-SSR1	(TA)_11_^a^
SSR2	HAMP2 in LG11 (ID: 100698871) 5′end 3.8 kb	OnHAMP2-SSR2	(CAGG)_6_
SSR3	HAMP2 in LG11 (ID: 100698871) 5′end 3.8 kb	OnHAMP2-SSR3	(GT)_13_
SSR4	HAMP2 in LG11 (ID: 100698871) 3′end 4.3 kb	OnHAMP2-SSR4	(CTAC)_7_
SSR5	HAMP2 in LG11 (ID: 100698871) 3′end 4.7 kb	OnHAMP2-SSR5	(TG)_21_
SSR6	HAMP1 in LG11 (ID: 109204280) 5′end 2 kb	OnHAMP1a-SSR1	(GT)_41_
SSR7	HAMP1 in LG11 (ID: 109204280) 3′end 4.7 kb	OnHAMP1a-SSR2	(AC)_12_
SSR8	HAMP1 in LG11 (ID: 109204256) 5′end 1.3 kb	OnHAMP1b-SSR1	(TG)_22_
SSR9	HAMP1 in LG11 (ID: 109204256) 5′end 1.3 kb	OnHAMP1b-SSR2	(T)_20_
SSR10	HAMP1 in LG11 (ID: 109204256) 3′end 7.6 kb	OnHAMP1b-SSR3	(TG)_10_
SSR11	HAMP4 in LG11 (ID: 109204092) 3′end 1 kb	OnHAMP4a-SSR	(CA)_29_
SSR12	HAMP1 in LG11 reverse strand (ID: 109204255) 5′end 2 kb	OnHAMP1c-SSR1	(TG)_27_
SSR13	HAMP1 in LG11 reverse strand (ID: 109204255) 5′end 6.3 kb	OnHAMP1c-SSR2	(ATTC)_7_
SSR14	HAMP1 in LG11 reverse strand (ID: 100534415) 3′end 6.2 kb	OnHAMP1d-SSR1	(TG)_22_
SSR15	HAMP1 in LG11 reverse strand (ID: 100534415) 3′end 750 bp	OnHAMP1d-SSR2	(AC)_13_
SSR16	HAMP1 in LG11 reverse strand (ID: 100534415) intron2	OnHAMP1d-SSR3	(T)_23_
SSR17	HAMP1 in LG11 (ID: 109204285) 3′end 4.2 kb	OnHAMP1g-SSR	(TG)_15_
SSR18	PGRN2 in LG22 (ID:100692931) PGRN2 3′end 2 kb	OnPGRN-SSR1	(TG)_10_
SSR19	PGRN1 in LG22 (ID:100534477) PGRN1 5′end 8.2 kb	OnPGRN-SSR2	(TTGA)_16_
SSR20	PGRN1 in LG22 (ID:100693478) PGRN1d 5′end 1.5 kb	OnPGRN-SSR3	(A)_42_
SSR21	PGRN1 in LG22 (ID:100693478) PGRN1d 3′end 3.1 kb	OnPGRN-SSR4	(TGTT)_10_
SSR22	TP4 in LG15 (ID:100698360) TP4 5′end 5 kb	OnTP4-SSR	(GAAAA)_6_

a. The number indicates repeat numbers of individual SSR identified in the Nile tilapia genome.

**Table 2:** Correlation analysis of SSRs and survival rate by chi-square test in strain A. Total of 11 loci, 10 of the 11 SSRs (except SSR7) that are statistically significant (*p* < 0.05). Df means degrees of freedom.

Locus	Value	df	Asymptotic Significance	Count Less than 5^a^	Minimum Expected Count
SSR2	40.463	9	<0.001	8 (40.0%)	0.32
SSR4	41.714	9	<0.001	11 (55.0%)	0.30
SSR5	31.947	16	0.01 0	21 (61.8%)	0.29
SSR7	2.2220	3	0.537	6 (75.0%)	0.30
SSR8	38.461	12	<0.001	16 (61.5.0%)	0.34
SSR14	45.794	7	<0.001	10 (62.5%)	0.29
SSR17	49.74	6	<0.001	8 (57.1%)	0.30
SSR18	51.019	10	<0.001	11 (50.0%)	0.28
SSR19	55.195	15	<0.001	23 (71.9%)	0.37
SSR21	74.447	23	<0.001	34 (70.8%)	0.28
SSR22	25.452	1	<0.001	0 (0.0%)	6.16

a. The genotypes with a count of less than five in both dead and alive groups. Red color = a statistically significant difference between genotype and dead or alive groups (*p* < 0.05).

**Table 3:** Correlation analysis of SSRs and survival rate by chi-square test in strain B. Total of 11 loci, only 1 of the 11 SSR (SSR5) that is statistically significant (*p* < 0.05). Df means degrees of freedom.

Locus	Value	df	Asymptotic Significance	Count Less than 5^a^	Minimum Expected Count
SSR2	3.077	5	0.688	5 (41.7%)	0.19
SSR4	5.815	5	0.325	2 (16.7%)	2.93
SSR5	54.563	31	0.006	55 (85.9%)	0.28
SSR7	15.109	11	0.178	15 (62.5%)	0.28
SSR8	2.11	8	0.977	12 (66.7%)	0.26
SSR14	27.267	28	0.504	52 (89.7%)	0.27
SSR17	23.905	26	0.581	45 (83.3%)	0.28
SSR18	4.987	9	0.835	9 (45.0%)	0.30
SSR19	19.739	20	0.474	31 (73.8%)	0.33
SSR21	2.57	7	0.922	5 (31.3%)	0.27
SSR22	1.912	3	0.591	5 (62.5%)	0.28

a. The genotypes with a count of less than five in both dead and alive groups. Red color = a statistically significant difference between genotype and dead or alive groups (*p* < 0.05).

**Table 4:** Correlation analysis of SSRs and survival rate by chi-square test in strain N2. Total of 11 loci, 3 of the 11 SSRs (SSR2, SSR14, and SSR22) that are statistically significant (*p* < 0.05). Df means degrees of freedom.

Locus	Value	df	Asymptotic Significance	Count Less than 5^a^	Minimum Expected Count
SSR2	21.079	4	<0.001	3 (30.0%)	1.27
SSR4	1.978	2	0.372	0 (0.0%)	5.42
SSR5	5.01	5	0.415	7 (58.3%)	0.42
SSR7	4.242	10	0.936	12 (54.5%)	0.21
SSR8	2.423	2	0.298	1 (16.7%)	3.40
SSR14	12.938	6	0.044	6 (42.9%)	0.63
SSR17	15.342	15	0.427	26 (81.3%)	0.17
SSR18	3.181	4	0.527	5 (50.0%)	0.21
SSR19	16.142	11	0.136	15 (62.5%)	0.24
SSR21	5.367	8	0.718	8 (44.4%)	0.21
SSR22	6.325	2	0.042	1 (16.7%)	3.81

a. The genotypes with a count of less than five in both dead and alive groups. Red color = a statistically significant difference between genotype and dead or alive groups (*p* < 0.05).

**Table 5:** The confusion matrix of the training and testing sets. There were 492 samples in the training set. True positive (TP) is 242; true negative (TN) is 181; false positive (FP) is 4; false negative (FN) is 65. In the testing set, there was a total of 39 samples. TP is 26; TN is 7; FP is 1; FN is 5.

	Training		Testing	
	Positive Prediction	Negative Prediction	Positive Prediction	Negative Prediction
Actual positive	242	65	26	5
Actual negative	4	181	1	7

**Table 6:** The validation index of the training and validation sets. All validation indices were calculated through the four parameters of TP, FP, TN, and FN. In addition to the FPR, FDR, and FNR, most of the validation indices are higher than 0.7. Only NPV and MCC in the testing set are lower than 0.7.

Measure	Training	Testing	Derivations
Sensitivity	0.7883	0.8387	TPR = TP/ (TP + FN)
Specificity	0.9891	0.8750	SPC = TN/ (FP + TN)
Precision	0.9918	0.9630	PPV = TP/ (TP + FP)
Negative predictive value (NPV)	0.7368	0.5833	NPV = TN/ (TN + FN)
False positive rate (FPR)	0.0109	0.1250	FPR = FP/ (FP + TN)
False discovery rate (FDR)	0.0082	0.0370	FDR = FP/ (FP + TP)
False negative rate (FNR)	0.2117	0.1613	FNR = FN/ (FN + TP)
Accuracy	0.8635	0.8462	ACC = (TP + TN)/ (P + N)
*F_1_* Score	0.8784	0.8966	*F_1_* = 2TP/(2TP + FP + FN
Matthews correlation coefficient (MCC)	0.7526	0.6244	TP × TN − FP × FN/sqrt((TP + FP) × (TP + FN) × (TN + FP) × (TN + FN))

## Data Availability

The datasets presented in this study can be found in online repositories. De novo transcriptome assembly of NT1 was submitted to the NCBI short read archive database (accession numbers: SRR14141863 and SRR14141864).

## Supplementary Materials

The following are available online at https://www.mdpi.com/article/10.3390/genes13010099/s1. Figure S1. The receiver operating characteristic curve (ROC curve) of the training and testing sets. Table S1: KOG orthologous group clustering analysis. Table S2: Distribution of the top 50 KEGG pathways and annotations of the NT1 strain. Table S3: GO annotation distribution of NT1. Table S4: The differential gene expression of *hepcidin*-, *progranulin*-, and *piscidin*-related transcripts after *S. iniae* infection. Table S5: Primer list. Table S6: *Streptococcus iniae* challenge after 14 days. Table S7. The allele frequencies of the disease-resistance-associated microsatellites. Table S8: The results of heterozygosity, *f*-statistics, and polymorphism by population for codominant data. Table S9: Pairwise population *F_ST_* values and estimates of *N_m_*. Table S10: The genotypes with count, % within group, and % within genotype in the dead and alive group. Table S11: The different combinations of genotypes and the predictive results of the new *Streptococcus*-resistant groups (F_1_) through SVM predictive model. Attachment 1: The raw data and predictive results of the predictive model.
