# Supplementary material for: Development of Disease-Resistance-Associated Microsatellite DNA Markers for Selective Breeding of Tilapia (Oreochromis spp.) Farmed in Taiwan
Source: Genes (Basel). 2021 Dec 31;13(1):99. doi: 10.3390/genes13010099 (PMC8774982; doi:10.3390/genes13010099)
Supplement: Supplementary file 1 [file genes-13-00099-s001.zip › genes-1535865-supplementary.pdf]

Supplementary Materials

Supplementary Materials: The following are available online at. Figure S1. The receiver operating characteristic curve (ROC curve) of the training and testing sets. Table S1: KOG orthologous group clustering analysis. Table S2: Distribution of the top 50 KEGG pathways and annotations of the NT1 strain. Table S3: GO annotation distribution of NT1. Table S4: The differential gene expression of *hepcidin-*, *progranulin-*, and *piscidin-* related transcripts after *S. iniae* infection. Table S5: Primer list. Table S6: *Streptococcus iniae* challenge after 14 days. Table S7. The alleles frequency of the disease-resistance-associated microsatellites. Table S8: The results of heterozygosity, *f*-statistics, and polymorphism by population for codominant data. Table S9: Pairwise population F_{ST} values and estimates of N_m. Table S10: The genotypes with count, % within group, and % within genotype in the dead and alive group. Table S11: The different combinations of genotypes and the predictive results of the new *Streptococcus*-resistant groups (F_1) through SVM predictive model. Attachment 1: The raw data and predictive results of the predictive model.

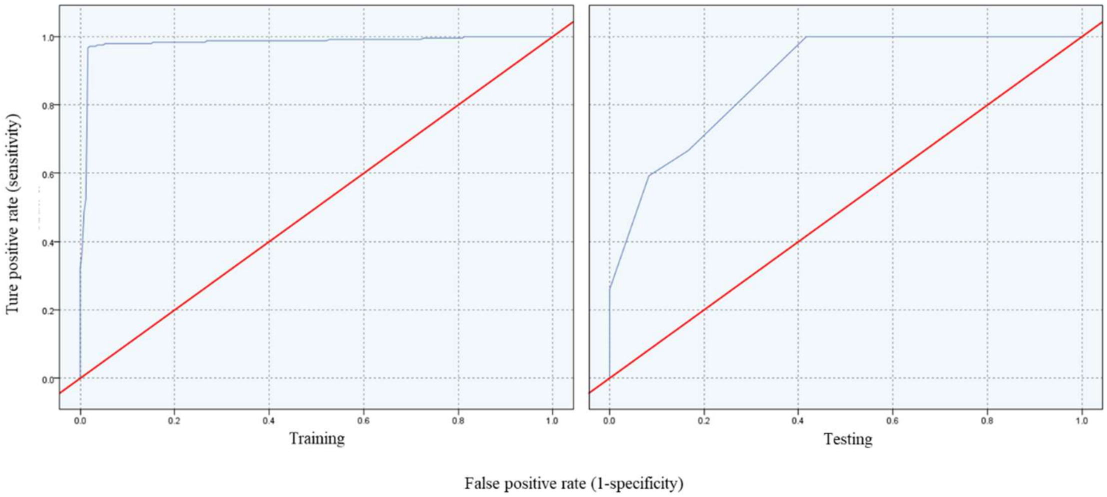

Figure S1. The receiver operating characteristic curve (ROC curve) of the training and testing sets. The x-axis is the false positive rate (1 − specificity) and the y-axis is the true positive rate (sensitivity). The blue line is the ROC curve and the red line is the diagonal line. The area under curve (AUC) is 0.983 in the training set and 0.849 in the testing set.

Table S1. KOG orthologous group clustering analysis. A total of 59,393 transcripts were annotated to 25 KOG orthologous groups.

KOG Description	Count	Percentage
**Information storage and processing**		
Translation, ribosomal structure, and biogenesis	938	1.45%
RNA processing and modification	1470	2.27%
Transcription	3316	5.12%
Replication, recombination, and repair	907	1.40%
Chromatin structure and dynamics	679	1.05%
**Cellular processing and signaling**		
Cell cycle control, cell division, chromosome partitioning	1163	1.80%
Nuclear structure	190	0.29%
Defense mechanisms	2415	3.73%
Signal transduction mechanisms	11789	18.20%
Cell wall/membrane/envelope biogenesis	387	0.60%
Cell motility	293	0.45%
Cytoskeleton	2688	4.15%
Extracellular structures	1429	2.21%
Intracellular trafficking, secretion, and vesicular transport	2797	4.32%

Posttranslational modification, protein turnover, chaperones	4294	6.63%
**Metabolism**		
Energy production and conversion	1035	1.60%
Carbohydrate transport and metabolism	1361	2.10%
Amino acid transport and metabolism	1513	2.34%
Nucleotide transport and metabolism	704	1.09%
Coenzyme transport and metabolism	305	0.47%
Lipid transport and metabolism	1805	2.79%
Inorganic ion transport and metabolism	1831	2.83%
Secondary metabolites biosynthesis, transport and catabolism	938	1.45%
**Poorly characterized**		
General function prediction only	10870	16.78%
Function unknown	9665	14.92%

Table S2. Distribution of the top 50 KEGG pathways and annotations of the NT1 strain. The table presents the top 50 pathways, K-number frequencies, and *p* value. A total of 22,690 transcripts were mapped to 377 KEGG pathways.

Pathway name	K-number frequencies	*p* value
Epstein–Barr virus infection	136 out of 4454	0.001
Endocytosis	170 out of 4454	0.002
RNA transport	125 out of 4454	0.002
Protein processing in endoplasmic reticulum	124 out of 4454	0.002
Viral carcinogenesis	124 out of 4454	0.002
Ribosome	119 out of 4454	0.003
Spliceosome	113 out of 4454	0.004
Tight junction	98 out of 4454	0.008
Lysosome	95 out of 4454	0.009
Autophagy—animal	94 out of 4454	0.009
Apoptosis	94 out of 4454	0.009
Fluid shear stress and atherosclerosis	87 out of 4454	0.013
Oxidative phosphorylation	105 out of 4454	0.033
Ribosome biogenesis in eukaryotes	65 out of 4454	0.040
RNA degradation	58 out of 4454	0.057
PI3K–Akt signaling pathway	203 out of 4454	0.060
Cell cycle—yeast	57 out of 4454	0.060
mRNA surveillance pathway	55 out of 4454	0.066
Adherens junction	55 out of 4454	0.066
Mitophagy—animal	53 out of 4454	0.073
ECM–receptor interaction	53 out of 4454	0.073
Non-alcoholic fatty liver disease (NAFLD)	115 out of 4454	0.075
Epithelial cell signaling in *Helicobacter pylori* infection	50 out of 4454	0.085
Renal cell carcinoma	50 out of 4454	0.085
Autophagy—yeast	49 out of 4454	0.089
Ubiquitin mediated proteolysis	109 out of 4454	0.092
Carbon metabolism	80 out of 4454	0.093
Thyroid hormone signaling pathway	80 out of 4454	0.093
Lysine degradation	48 out of 4454	0.094
Biosynthesis of amino acids	47 out of 4454	0.098
B cell receptor signaling pathway	47 out of 4454	0.098
Necroptosis	78 out of 4454	0.101
PPAR signaling pathway	45 out of 4454	0.109
Pyrimidine metabolism	75 out of 4454	0.114

Inositol phosphate metabolism	44 out of 4454	0.114
Longevity regulating pathway—worm	43 out of 4454	0.120
Purine metabolism	127 out of 4454	0.122
Renin secretion	42 out of 4454	0.126
NF-kappa B signaling pathway	72 out of 4454	0.128
T cell receptor signaling pathway	72 out of 4454	0.128
TNF signaling pathway	72 out of 4454	0.128
Valine, leucine, and isoleucine degradation	41 out of 4454	0.133
Platinum drug resistance	41 out of 4454	0.133
Cell cycle	97 out of 4454	0.139
Apoptosis—fly	40 out of 4454	0.139
Synaptic vesicle cycle	40 out of 4454	0.139
Sphingolipid signaling pathway	69 out of 4454	0.144
Toxoplasmosis	69 out of 4454	0.144
Nucleotide excision repair	39 out of 4454	0.146
Peroxisome	68 out of 4454	0.149

Table S3. GO annotation distribution of NT1. A total of 22,788 transcripts were annotated to 56 GO functional groups ($p < 0.01$).

Gene term name	Transcript ID frequency	*p* value
oxidoreductase activity	1147 out of 22690	3E-10
coenzyme binding	418 out of 22690	7E-07
iron ion binding	221 out of 22690	9E-07
oxidoreductase activity, acting on peroxide as acceptor	39 out of 22690	1E-05
oxidoreductase activity, acting on paired donors, with incorporation or reduction of molecular oxygen	197 out of 22690	5E-05
antioxidant activity	83 out of 22690	7E-05
translation factor activity, RNA binding	108 out of 22690	1E-04
phosphofructokinase activity	67 out of 22690	2E-04
carbohydrate kinase activity	67 out of 22690	2E-04
cyclase regulator activity	20 out of 22690	2E-04
cyclase activator activity	20 out of 22690	2E-04
guanylate cyclase regulator activity	20 out of 22690	2E-04
guanylate cyclase activator activity	20 out of 22690	2E-04
thiamine pyrophosphate binding	33 out of 22690	6E-04
cyclin-dependent protein serine/threonine kinase activity	24 out of 22690	8E-04
cyclin-dependent protein kinase activity	24 out of 22690	8E-04
enzyme inhibitor activity	214 out of 22690	8E-04
endopeptidase inhibitor activity	147 out of 22690	0.001
endopeptidase regulator activity	147 out of 22690	0.001
cofactor binding	340 out of 22690	0.001
protein kinase regulator activity	33 out of 22690	0.001
heme binding	211 out of 22690	0.002
oxidoreductase activity, acting on the CH-NH group of donors	39 out of 22690	0.002
threonine-type endopeptidase activity	24 out of 22690	0.002
threonine-type peptidase activity	24 out of 22690	0.002
NAD binding	162 out of 22690	0.002
structural constituent of cytoskeleton	51 out of 22690	0.003
6-phosphofructokinase activity	15 out of 22690	0.003
oxidoreductase activity, acting on the CH-NH group of donors, NAD or NADP as acceptor	38 out of 22690	0.003

protein kinase inhibitor activity	21 out of 22690	0.003
hydrolase activity, hydrolyzing N-glycosyl compounds	21 out of 22690	0.003
kinase inhibitor activity	21 out of 22690	0.003
peroxiredoxin activity	21 out of 22690	0.003
peroxidase activity	18 out of 22690	0.003
protein serine/threonine kinase inhibitor activity	18 out of 22690	0.003
phosphatidylinositol phosphate binding	11 out of 22690	0.003
peptidase inhibitor activity	162 out of 22690	0.003
peptidase regulator activity	162 out of 22690	0.003
oxidoreductase activity, acting on the CH-OH group of donors, NAD or NADP as acceptor	177 out of 22690	0.003
intramolecular transferase activity	35 out of 22690	0.003
fatty-acyl-CoA binding	37 out of 22690	0.004
vitamin binding	110 out of 22690	0.004
NAD+ binding	25 out of 22690	0.004
glycerone kinase activity	14 out of 22690	0.004
oxidoreductase activity, acting on the CH-CH group of donors, oxygen as acceptor	14 out of 22690	0.004
translation initiation factor activity	61 out of 22690	0.004
metalloendopeptidase inhibitor activity	10 out of 22690	0.005
6-phosphofructo-2-kinase activity	52 out of 22690	0.006
oxidoreductase activity, acting on CH-OH group of donors	179 out of 22690	0.006
cyclin-dependent protein serine/threonine kinase regulator activity	16 out of 22690	0.007
oxidoreductase activity, acting on diphenols and related substances as donors	16 out of 22690	1E-04
structural constituent of myelin sheath	16 out of 22690	2E-04
acyl-CoA oxidase activity	13 out of 22690	2E-04
ribosome binding	13 out of 22690	2E-04
oxidoreductase activity, acting on NAD(P)H, quinone or similar compound as acceptor	45 out of 22690	2E-04
phosphatidylinositol-3-phosphate binding	9 out of 22690	2E-04

Table S4. The differential gene expression of hepcidin-, progranulin-, and piscidin- related transcripts after *S. iniae* infection.

Transcript ID	Length	Description	FPKM NT1	FPKM NT1S
NT1_GG17866丨c0_g1_i1	387	PREDICTED: hepcidin-like	0.65	2.28
NT1_GG18280丨c0_g1_i1	226	PREDICTED: hepcidin-like	0	2.71
NT1_GG43388丨c0_g1_i1	420	PREDICTED: hepcidin-like	0.19	2.14
NT1_GG43388丨c0_g1_i2	411	PREDICTED: hepcidin-like	0.58	2.84
NT1_GG33569丨c2_g1_i2	1902	PREDICTED: progranulin-like	12.3	9.46
NT1_GG33570丨c1_g2_i4	1450	progranulin precursor	41.25	37.95
NT1_GG22982丨c0_g1_i4	722	PREDICTED: piscidin-like	13.7	29.29

Table S5. Primer list.

SSR	SSR name	Primer sequence
SSR1	OnHAMP2-SSR1	F: gagcacgaggacactgaCCACACAATCAACACACTGGTA<br>R: TCAAACAGAAACAGGGACACAC
SSR2	OnHAMP2-SSR2	F: gagcacgaggacactgaGCACAGACACAGTAACACATGC<br>R: ACTCCCTGGTACATGCTTCCTA
SSR3	OnHAMP2-SSR3	F: gagcacgaggacactgaTAGGAAGCATGTACCAGGGAGT<br>R: AAAATCACTCAACCGTGTCCTT
SSR4	OnHAMP2-SSR4	F: gagcacgaggacactgaCACCACTGTCAACTGGCTAATG<br>R: GTTACCTTCTTGATACCGCAGG
SSR5	OnHAMP2-SSR5	F: gagcacgaggacactgaCTTTGGGTAGAGGAACACTCCA<br>R: TGCAGGTCAATAGCAATACCAC

SSR6	OnHAMP1a-SSR1	F: gagcacgaggacactgaCAGTGGGTGTTTGTTCCTTACA<br>R: TAGTAGGCTTTGTGTGCATTCC
SSR7	OnHAMP1a-SSR2	F: gagcacgaggacactgaCCACACGCACTCTCACCA<br>R: CTGGTAGCCTTGACCCAGTTT
SSR8	OnHAMP1b-SSR1	F: gagcacgaggacactgaCCCCATAGCACTCCTTTTATTG<br>R: TCATTTGGAGGTGTTTTCACAG
SSR9	OnHAMP1b-SSR2	F: gagcacgaggacactgaGCGCTGTATAAGATTCCCGTTA<br>R: GGAAACACAAGAGACATGAGCA
SSR10	OnHAMP1b-SSR3	F: gagcacgaggacactgaGTTTGTAGCTTAACCCATTCGC<br>R: TGCCTTTGTTTAGATGAACTGC
SSR11	OnHAMP4a-SSR	F: gagcacgaggacactgaAAGTGTCGTTCCACCCACAT<br>R: ACAGAGTGTTCTGGCTTTCACA
SSR12	OnHAM1c-SSR1	F: gagcacgaggacactgaGTGCATTACAGAGTGTTCTGGC<br>R: GTCTGGAGCCAAGTGTCGTT
SSR13	OnHAM1c-SSR2	F: gagcacgaggacactgaTTAAAATGGGCTCAGGAGAAAG<br>R: ACACACATAGATTCATCCGCAC
SSR14	OnHAMP1d-SSR1	F: gagcacgaggacactgaTCGGAATTGAGCATTAAGACCT<br>R: GGTTCCCCATAGAACTCCTTTT
SSR15	OnHAMP1d-SSR2	F: gagcacgaggacactgaCAACAAGTGAAGCGACCATATT<br>R: TCACACACAAGCAGGTCAATTA
SSR16	OnHAMP1d-SSR3	F: gagcacgaggacactgaGTGGGAAACACAAGAGACATGA<br>R: CAGGGCTGAGATAGATTTTGGT
SSR17	OnHAMP1g-SSR	F: gagcacgaggacactgaGGTTCCCCATAGAACTCTTTT<br>R: TCGGAATTGAGCATTAAGACCT
SSR18	OnPGRN-SSR1	F: gagcacgaggacactgaTGAAAGGAGAACTGAAGCACAA<br>R: TCTGGATTTGATGGAATGTCTG
SSR19	OnPGRN-SSR2	F: gagcacgaggacactgaGGACCCTGAATCTTCCCTTAGT<br>R: GGCTCTCTCTCTTGAGTTGGAC
SSR20	OnPGRN-SSR3	F: gagcacgaggacactgaCTCAGGACTTGCTGTGATGATT<br>R: TGTAGCGGTTTCATTTGTTCAC
SSR21	OnPGRN-SSR4	F: gagcacgaggacactgaACTAGCAGATGATAAATGCGCC<br>R: ATGCCCAGACCTAAATCCTACA
SSR22	OnTP4-SSR	F: gagcacgaggacactgaTGTATGTAGCCGAAGTAGCCAA<br>R: CCTTGCAGTGAACCCTTTGTAT

gagcacgaggacactga is the adapter sequences.

Table S6. *Streptococcus iniae* challenge after 14 days. Total number of A is 200; B is 198; N2 is 197.

	A	B	N2
Dose	$2*10^6$	$6*10^5$	$6.5*10^5$
Day 0	0	0	0
Day 1	45	44	130
Day 2	67	29	20
Day 3	12	18	6
Day 4	6	16	1
Day 5	2	6	0
Day 6	1	25	0
Day 7	4	1	0
Day 8	3	1	0
Day 9	1	1	0
Day 10	1	4	0
Day 11	2	0	0

Day 12	0	0	0
Day 13	0	0	0
Day 14	0	0	0
Residual amount	58	53	40
Lethal dose	71.287	73.232	79.695

Table S7. The alleles frequency of the disease-resistance-associated microsatellites.

SSR	pop	A	B	C	D	E	F	G	H	I	J	K	L	M	N	O	P	Q	R
SSR2	A	0.180	0.243	0.459	0.052	0.019	0.047												
	B	0.000	0.677	0.245	0.078	0.000	0.000												
	N2	0.000	0.333	0.556	0.000	0.016	0.095												
SSR4	A	0.005	0.128	0.547	0.039	0.281													
	B	0.372	0.000	0.266	0.361	0.000													
	N2	0.000	0.000	0.568	0.432	0.000													
SSR5	A	0.000	0.191	0.000	0.249	0.336	0.027	0.000	0.000	0.000	0.000	0.000	0.000	0.087	0.109	0.000	0.000	0.000	0.000
	B	0.000	0.000	0.247	0.000	0.000	0.008	0.013	0.021	0.023	0.148	0.039	0.104	0.000	0.000	0.029	0.026	0.294	0.047
	N2	0.353	0.000	0.626	0.000	0.000	0.010	0.005	0.000	0.000	0.000	0.005	0.000	0.000	0.000	0.000	0.000	0.000	0.000
SSR7	A	0.000	0.003	0.495	0.503	0.000	0.000	0.000											
	B	0.000	0.031	0.414	0.247	0.240	0.036	0.031											
	N2	0.000	0.063	0.464	0.201	0.117	0.156	0.000											
SSR8	A	0.012	0.315	0.106	0.015	0.006	0.124	0.424	0.000	0.000	0.000	0.000	0.000						
	B	0.000	0.000	0.000	0.000	0.067	0.326	0.000	0.090	0.152	0.045	0.174	0.146						
	N2	0.000	0.000	0.000	0.806	0.194	0.000	0.000	0.000	0.000	0.000	0.000	0.000						
SSR14	A	0.000	0.014	0.657	0.000	0.078	0.000	0.005	0.211	0.000	0.022	0.014	0.000	0.000	0.000				
	B	0.098	0.005	0.142	0.424	0.000	0.055	0.109	0.003	0.055	0.038	0.011	0.027	0.016	0.016				
	N2	0.000	0.000	0.482	0.161	0.000	0.289	0.000	0.023	0.000	0.000	0.000	0.044	0.000	0.000				
SSR17	A	0.747	0.005	0.055	0.003	0.156	0.016	0.018	0.000	0.000	0.000	0.000	0.000	0.000	0.000	0.000	0.000	0.000	
	B	0.156	0.419	0.000	0.110	0.000	0.032	0.000	0.102	0.043	0.065	0.043	0.016	0.013	0.000	0.000	0.000	0.000	
	N2	0.478	0.031	0.000	0.000	0.012	0.000	0.000	0.000	0.176	0.000	0.059	0.000	0.000	0.201	0.009	0.028	0.006	
SSR18	A	0.162	0.553	0.136	0.144	0.005													
	B	0.377	0.187	0.172	0.265	0.000													
	N2	0.295	0.670	0.035	0.000	0.000													
SSR19	A	0.091	0.436	0.037	0.139	0.057	0.010	0.230	0.000	0.000	0.000								
	B	0.000	0.250	0.148	0.207	0.000	0.043	0.179	0.170	0.003	0.000								
	N2	0.000	0.191	0.000	0.165	0.015	0.180	0.257	0.000	0.000	0.191								
SSR21	A	0.074	0.063	0.005	0.008	0.330	0.181	0.234	0.011	0.093	0.000								
	B	0.413	0.379	0.003	0.203	0.003	0.000	0.000	0.000	0.000	0.000								
	N2	0.291	0.333	0.000	0.161	0.212	0.000	0.000	0.000	0.000	0.003								
SSR22	A	0.946	0.054	0.000															
	B	0.961	0.008	0.031															
	N2	0.325	0.675	0.000															

Table S8. The results of heterozygosity, *f*-statistics, and polymorphism by population for codominant data.

**Population**	**N**	**Na**	**Ne**	**I**	**Ho**	**He**	**uHe**	**F**	**Pic**

A	Mean	181.300	6.200	3.015	1.247	0.634	0.633	0.635	-0.042	0.583
	SE	4.271	0.512	0.314	0.104	0.051	0.039	0.039	0.116	0.136
B	Mean	165.000	7.000	3.907	1.485	0.591	0.718	0.720	0.167	0.675
	SE	10.817	1.135	0.368	0.126	0.060	0.033	0.033	0.084	0.122
N2	Mean	179.600	4.600	2.786	1.079	0.656	0.591	0.592	-0.057	0.527
	SE	6.021	0.653	0.345	0.125	0.095	0.048	0.048	0.109	0.170

N = Number of alleles. N_a = No. of Different Alleles. N_e = No. of Effective Alleles. I = Shannon's Information Index. H_o = Observed Heterozygosity H_e = Expected Heterozygosity. uH_e = Unbiased Expected Heterozygosity. F = Fixation Index. PIC = polymorphic information content.

Table S9. Pairwise population F_{ST} values and estimates of N_m.

Pop1	Pop2	Fst	Nm	#Pop1	#Pop2
A	B	0.114	1.950	192	192
A	N2	0.102	2.191	192	192
B	N2	0.098	2.294	192	192

F_{ST} = genetic differentiation coefficient = (H_t - Mean H_e) / H_t; N_m = number of migrants per generation = [(1 / F_{ST}) - 1] / 4.

Table S10. The genotypes with count, % within Group, and % within Genotype in the dead and alive group.

SSR	Genotype	Pop	Count	Dead group Count	Dead group % within Group	Dead group % within Genotype	Alive group count	Alive group % within Group	Alive group % within Genotype
SSR2	AC	A	24	22	17.9	91.7	2	3.4	8.3
	AD	A	18	18	14.6	100	0	0	0
	BC	A	49	24	19.5	49	25	43.1	51
		B	40	30	26.3	75	10	37	25
		N2	94	78	52.3	83	16	40	17
	CC	A	38	17	13.8	44.7	21	36.5	55.3
		B	11	8	7	72.7	3	11.1	27.3
		N2	40	23	15.4	57.5	17	42.5	42.5
	CF	N2	36	34	22.8	94.4	2	5	5.6
SSR4	AC	A	2	2	1.5	100	0	0	0
		B	35	30	22.2	85.7	5	10.2	14.3
	BC	A	39	29	21.6	74.4	10	17.2	25.6
	CC	A	51	45	33.6	88.2	6	10.4	11.8
		B	11	9	6.7	81.8	2	4.1	18.2
		N2	52	43	28.3	82.7	9	22.5	17.4
	DD	A	1	1	0.7	100	0	0	0
		B	18	11	8.1	61.1	7	14.3	38.9
		N2	26	18	11.8	69.2	8	20	30.8
	DE	A	8	1	0.7	12.5	7	12.1	87.5
SSR5	EF	A	2	0	0	0	2	3.8	100
	MN	A	22	21	16.3	95.5	1	1.9	4.5
	LQ	B	20	19	13.8	95	1	1.9	5
	PQ	B	5	0	0	0	5	9.3	100
SSR7	CD	A	187	129	96.3	69	58	100	31

		B	69	57	41	82.6	12	22.6	17.4
	CE	B	68	42	30.2	61.8	26	49.1	38.2
		N2	36	30	19.7	83.3	6	15	16.7
	EE	B	1	0	0	0	1	1.9	100
	EG	B	5	2	1.4	40	3	5.7	60
SSR8	BF	A	13	12	10.6	92.3	1	1.8	7.7
	CF	A	25	8	7.1	32	17	29.8	68
	EF	A	18	17	15	94.4	1	1.8	5.6
SSR14	BC	A	5	0	0	0	5	9.3	100
		B	2	2	1.5	100	0	0	0
	CC	A	70	42	32.3	60	28	51.9	40
		B	15	8	6	53.3	7	14.3	46.7
		N2	25	18	11.8	72	7	17.5	28
	CD	B	9	7	5.2	77.8	2	4.1	22.2
		N2	53	46	30.3	86.8	7	17.5	13.2
	CE	A	29	14	10.8	48.3	15	27.8	51.7
	CH	A	65	60	46.2	92.3	5	9.3	7.7
		N2	3	3	2	100	0	0	0
	HI	A	7	7	5.4	100	0	0	0
	FH	N2	6	2	1.3	33.3	4	10	66.7
SSR17	AC	A	21	3	2.2	14.3	18	31	85.7
	AE	A	47	43	32.1	91.5	4	6.9	8.5
		N2	2	2	1.5	100	0	0	0
	AI	N2	43	36	26.9	c.7	7	25	16.3
	AK	N2	13	7	5.2	53.8	6	21.4	46.2
SSR18	AA	A	7	1	0.7	14.3	6	11.5	85.7
		B	40	29	30.9	72.5	11	27.5	27.5
		N2	44	34	23	77.3	10	25	22.7
	AC	A	5	0	0	0	5	9.6	100
		B	8	6	6.4	54.5	2	5	25
	BB	A	57	47	34.6	82.5	10	19.2	17.5
		B	18	13	13.8	72.2	5	12.5	27.8
		N2	114	91	61.5	79.8	23	57.5	20.2
	BC	A	24	21	15.4	87.5	3	5.8	12.5
		B	1	0	0	0	1	2.5	100
		N2	1	1	0.7	100	0	0	0
	BD	A	34	29	21.3	85.3	5	9.6	14.7
		B	11	9	9.6	81.8	2	5	18.2
	CC	A	6	6	4.4	100	0	0	0
		B	17	12	12.8	70.6	5	12.5	29.4
		N2	6	6	4.1	100	0	0	0
SSR19	BB	A	47	17	17.5	36.2	30	53.6	63.8
		B	14	7	6.4	50	7	13.2	50

	BD	A	3	3	3.1	100	0	0	0
		B	15	10	9.2	66.7	5	9.4	33.3
		N2	15	9	8.7	60	6	18.2	40
	BF	N2	18	17	16.5	94.4	1	3	5.6
	BG	A	26	24	24.7	92.3	2	3.6	7.7
		B	14	11	10.1	78.6	3	5.7	21.4
	DD	B	8	3	2.8	37.5	5	9.4	62.5
		N2	3	3	2.9	100	0	0	0
	EE	A	3	0	0	0	3	5.4	100
	GG	A	13	13	13.4	100	0	0	0
		B	3	2	1.8	66.7	1	1.9	33.3
SSR21	EE	A	29.6	22	16.8	81.5	7.6	9.8	18.5
	EF	A	23	11	8.4	47.8	12	23.5	52.2
	EG	A	19	18	13.7	94.7	1	2	5.3
	FF	A	10	0	0	0	10	19.6	100
	FG	A	9	8	6.1	88.9	1	2	11.1
	GG	A	14	14	10.7	100	0	0	0
	GI	A	14	12	9.2	85.7	2	3.9	14.3
SSR22	AA	A	165	124	96.9	75.2	41	71.9	24.8
		B	178	127	91.4	71.3	51	96.2	28.7
		N2	18	12	8.1	66.7	6	15	33.3
	AB	A	20	4	3.1	20	16	28.1	80
		B	3	3	2.2	100	0	0	0
		N2	87	64	43	73.6	23	57.7	26.4
	BB	N2	84	73	49	86.9	11	27.5	13.1

Blue color: relate to death. Red color: relate to survival.

Table S11. The different combinations of genotypes and the predictive results of the new *Streptococcus*-resistant groups (F_1) through SVM predictive model. Three strains were ANT1 (N = 96), AB (N = 55), and BB (N = 40). $S: The predicted value of target field. Here, the target is death (1). $SP: Probability of predicted value. $SP-value: Probability of each possible value of the flag, alive (0) or death (1).

Group	SSR2	SSR4	SSR7	SSR14	SSR18	SSR19	SSR21	SSR22	$S	$SP	$SP-1	$SP-0
ANT1	9	9	8	0	8	0	2	1	1	0.998	0.002	0.998
ANT1	9	7	8	0	8	0	2	1	1	0.997	0.003	0.997
ANT1	6	9	8	0	8	0	2	1	1	0.997	0.003	0.997
ANT1	9	7	8	0	8	0	2	1	1	0.997	0.003	0.997
ANT1	9	9	5	0	8	0	2	1	1	0.997	0.003	0.997
ANT1	9	9	5	0	8	0	2	1	1	0.997	0.003	0.997
ANT1	6	7	5	0	8	0	2	1	1	0.996	0.004	0.996
ANT1	6	7	5	0	8	0	2	1	1	0.996	0.004	0.996
ANT1	9	7	8	0	8	0	0	1	1	0.996	0.004	0.996
ANT1	9	7	5	0	8	0	2	1	1	0.994	0.006	0.994
ANT1	9	2	8	0	8	0	2	2	1	0.994	0.006	0.994
ANT1	9	7	5	0	8	0	2	1	1	0.994	0.006	0.994

ANT1	9	7	5	0	8	0	2	1	1	0.994	0.006	0.994
ANT1	6	2	5	0	8	0	2	1	1	0.994	0.006	0.994
ANT1	9	9	8	0	8	0	2	2	1	0.994	0.006	0.994
ANT1	9	2	8	0	8	0	2	2	1	0.994	0.006	0.994
ANT1	9	2	8	0	8	0	2	2	1	0.994	0.006	0.994
ANT1	9	9	8	0	8	0	2	2	1	0.994	0.006	0.994
ANT1	9	9	8	0	8	0	2	2	1	0.994	0.006	0.994
ANT1	9	2	8	0	8	0	2	2	1	0.994	0.006	0.994
ANT1	9	9	8	0	8	0	2	2	1	0.994	0.006	0.994
ANT1	6	2	8	0	8	0	2	1	1	0.993	0.007	0.993
ANT1	6	2	8	0	8	0	2	1	1	0.993	0.007	0.993
ANT1	9	7	8	0	8	0	2	2	1	0.993	0.007	0.993
ANT1	9	7	8	0	8	0	2	2	1	0.993	0.007	0.993
ANT1	9	7	5	0	8	0	0	1	1	0.99	0.01	0.99
ANT1	9	9	5	0	8	0	2	2	1	0.989	0.011	0.989
ANT1	9	9	5	0	8	0	2	2	1	0.989	0.011	0.989
ANT1	9	0	5	0	8	0	2	1	1	0.989	0.011	0.989
ANT1	9	0	5	0	8	0	2	1	1	0.989	0.011	0.989
ANT1	9	0	5	0	8	0	2	1	1	0.989	0.011	0.989
ANT1	6	0	5	0	8	0	2	1	1	0.989	0.011	0.989
ANT1	6	0	5	0	8	0	2	1	1	0.989	0.011	0.989
ANT1	6	0	5	0	8	0	2	1	1	0.989	0.011	0.989
ANT1	9	9	5	0	8	0	2	2	1	0.989	0.011	0.989
ANT1	9	0	5	0	8	0	2	1	1	0.989	0.011	0.989
ANT1	6	0	5	0	8	0	2	1	1	0.989	0.011	0.989
ANT1	0	2	5	0	8	0	2	1	1	0.989	0.011	0.989
ANT1	6	9	5	0	8	0	2	2	1	0.988	0.012	0.988
ANT1	6	9	5	0	8	0	2	2	1	0.988	0.012	0.988
ANT1	0	7	8	0	8	0	0	1	1	0.987	0.013	0.987
ANT1	9	7	5	0	8	0	2	2	1	0.985	0.015	0.985
ANT1	9	7	5	0	8	0	2	2	1	0.985	0.015	0.985
ANT1	9	7	5	0	8	0	2	2	1	0.985	0.015	0.985
ANT1	9	7	5	0	8	0	2	2	1	0.985	0.015	0.985
ANT1	6	9	8	0	8	0	2	2	1	0.984	0.016	0.984
ANT1	6	7	5	0	8	0	2	2	1	0.983	0.017	0.983
ANT1	6	7	5	0	8	0	2	2	1	0.983	0.017	0.983
ANT1	6	7	5	0	8	0	2	2	1	0.983	0.017	0.983
ANT1	6	2	5	0	8	0	2	2	1	0.982	0.018	0.982
ANT1	6	2	8	0	8	0	2	2	1	0.981	0.019	0.981
ANT1	6	2	8	0	8	0	2	2	1	0.981	0.019	0.981
ANT1	6	0	8	0	8	0	2	1	1	0.981	0.019	0.981
ANT1	6	0	8	0	8	0	2	1	1	0.981	0.019	0.981
ANT1	6	2	8	0	8	0	2	2	1	0.981	0.019	0.981

ANT1	6	2	8	0	8	0	2	2	1	0.981	0.019	0.981
ANT1	6	0	8	0	8	0	2	1	1	0.981	0.019	0.981
ANT1	9	0	5	0	8	0	2	2	1	0.98	0.02	0.98
ANT1	6	0	5	0	8	0	2	2	1	0.97	0.03	0.97
ANT1	6	0	5	0	8	0	2	2	1	0.97	0.03	0.97
ANT1	9	0	5	0	8	0	0	2	1	0.963	0.037	0.963
ANT1	6	0	8	0	8	0	2	2	1	0.96	0.04	0.96
ANT1	9	9	5	0	8	5	0	1	1	0.956	0.044	0.956
ANT1	9	9	5	0	8	5	0	1	1	0.956	0.044	0.956
ANT1	9	9	5	0	8	5	0	1	1	0.956	0.044	0.956
ANT1	9	7	8	0	8	5	0	1	1	0.949	0.051	0.949
ANT1	6	9	5	0	8	5	0	1	1	0.931	0.069	0.931
ANT1	9	9	5	0	8	5	2	2	1	0.924	0.076	0.924
ANT1	9	7	5	0	8	5	0	1	1	0.918	0.082	0.918
ANT1	9	7	5	0	8	5	0	1	1	0.918	0.082	0.918
ANT1	9	7	5	0	8	5	0	1	1	0.918	0.082	0.918
ANT1	9	2	8	0	8	5	0	2	1	0.915	0.085	0.915
ANT1	9	2	8	0	8	5	0	2	1	0.915	0.085	0.915
ANT1	6	0	5	0	8	0	0	2	1	0.91	0.09	0.91
ANT1	6	0	5	0	8	0	0	2	1	0.91	0.09	0.91
ANT1	9	7	8	0	8	5	0	2	1	0.908	0.092	0.908
ANT1	9	2	5	0	8	5	0	1	1	0.907	0.093	0.907
ANT1	9	7	5	0	0	5	0	2	1	0.903	0.097	0.903
ANT1	9	9	5	0	8	5	0	2	1	0.882	0.118	0.882
ANT1	9	9	5	0	8	5	0	2	1	0.882	0.118	0.882
ANT1	6	7	5	0	8	5	0	1	1	0.863	0.137	0.863
ANT1	9	7	5	0	8	5	0	2	1	0.85	0.15	0.85
ANT1	9	7	5	0	8	5	0	2	1	0.85	0.15	0.85
ANT1	6	2	5	0	8	5	0	1	1	0.825	0.175	0.825
ANT1	6	2	5	0	8	5	0	1	1	0.825	0.175	0.825
ANT1	9	0	5	0	8	5	0	2	1	0.794	0.206	0.794
ANT1	6	0	5	0	8	5	2	2	1	0.758	0.242	0.758
ANT1	6	0	5	0	8	5	2	2	1	0.758	0.242	0.758
ANT1	6	7	5	0	8	5	0	2	1	0.705	0.295	0.705
ANT1	6	2	5	0	8	5	0	2	1	0.691	0.309	0.691
ANT1	6	2	5	0	8	5	0	2	1	0.691	0.309	0.691
ANT1	6	2	5	0	8	5	0	2	1	0.691	0.309	0.691
ANT1	6	0	5	0	8	5	0	1	1	0.683	0.317	0.683
ANT1	6	7	8	0	8	5	0	2	1	0.65	0.35	0.65
ANT1	6	2	8	0	8	5	0	2	1	0.636	0.364	0.636
ANT1	6	0	8	0	8	5	0	2	0	0.571	0.571	0.429
AB	10	9	7	10	7	9	16	1	1	0.999	0.001	0.999
AB	10	9	7	10	7	9	16	1	1	0.999	0.001	0.999

AB	10	9	7	10	7	9	16	1	1	0.999	0.001	0.999
AB	10	9	7	10	7	25	16	1	1	0.998	0.002	0.998
AB	9	9	8	10	0	0	1	2	1	0.998	0.002	0.998
AB	10	8	7	10	7	0	16	1	1	0.997	0.003	0.997
AB	10	8	7	10	7	0	16	1	1	0.997	0.003	0.997
AB	10	9	7	10	11	0	4	2	1	0.996	0.004	0.996
AB	10	9	7	10	11	0	16	2	1	0.995	0.005	0.995
AB	9	10	7	10	7	0	16	2	1	0.995	0.005	0.995
AB	10	11	7	10	11	0	4	1	1	0.994	0.006	0.994
AB	10	9	7	10	7	9	16	2	1	0.992	0.008	0.992
AB	10	9	7	10	8	20	4	1	1	0.991	0.009	0.991
AB	9	9	7	10	7	9	16	1	1	0.99	0.01	0.99
AB	10	8	8	10	11	0	4	1	1	0.987	0.013	0.987
AB	9	10	7	10	7	9	16	1	1	0.985	0.015	0.985
AB	9	11	8	10	8	0	4	2	1	0.985	0.015	0.985
AB	10	8	7	10	7	9	16	1	1	0.983	0.017	0.983
AB	9	10	7	10	7	9	4	1	1	0.981	0.019	0.981
AB	10	8	7	10	8	0	4	2	1	0.977	0.023	0.977
AB	9	8	7	10	7	0	16	2	1	0.969	0.031	0.969
AB	9	11	7	10	0	0	1	2	1	0.965	0.035	0.965
AB	10	9	8	10	8	20	4	1	1	0.96	0.04	0.96
AB	9	11	7	10	8	0	4	1	1	0.959	0.041	0.959
AB	9	8	7	10	8	0	4	1	1	0.948	0.052	0.948
AB	10	9	8	10	8	20	4	2	1	0.948	0.052	0.948
AB	9	11	7	10	8	0	4	2	1	0.943	0.057	0.943
AB	10	10	7	10	0	7	1	1	1	0.932	0.068	0.932
AB	9	11	0	10	7	9	16	2	1	0.931	0.069	0.931
AB	9	11	7	10	7	9	16	1	1	0.931	0.069	0.931
AB	9	10	8	10	8	20	4	1	1	0.92	0.08	0.92
AB	9	10	8	10	8	20	4	1	1	0.92	0.08	0.92
AB	10	11	7	10	8	20	4	1	1	0.914	0.086	0.914
AB	10	10	7	10	11	4	16	2	1	0.897	0.103	0.897
AB	9	10	7	10	8	20	4	1	1	0.888	0.112	0.888
AB	9	10	7	10	8	20	4	1	1	0.888	0.112	0.888
AB	9	8	7	10	7	9	16	2	1	0.875	0.125	0.875
AB	9	10	8	10	11	4	4	2	1	0.87	0.13	0.87
AB	9	10	8	10	11	4	4	2	1	0.87	0.13	0.87
AB	10	9	8	10	0	7	1	2	1	0.863	0.137	0.863
AB	10	8	7	10	8	20	4	2	1	0.837	0.163	0.837
AB	9	11	7	10	11	4	4	1	0	0.763	0.763	0.237
AB	9	10	7	10	0	7	1	2	1	0.749	0.251	0.749
AB	9	10	7	10	0	7	1	2	1	0.749	0.251	0.749
AB	9	10	7	10	11	4	16	2	1	0.74	0.26	0.74

AB	10	8	8	10	11	4	4	2	1	0.706	0.294	0.706
AB	10	8	8	10	0	7	1	2	1	0.706	0.294	0.706
AB	9	11	7	10	0	7	1	2	0	0.655	0.655	0.345
AB	9	11	7	10	8	20	4	2	1	0.651	0.349	0.651
AB	10	8	7	10	11	4	4	2	1	0.65	0.35	0.65
AB	9	11	7	10	8	20	4	1	1	0.632	0.368	0.632
AB	9	8	7	10	8	20	16	2	1	0.625	0.375	0.625
AB	9	8	7	10	0	7	1	1	0	0.598	0.598	0.402
AB	10	8	8	10	11	4	1	1	1	0.582	0.418	0.582
AB	10	8	7	10	0	7	1	2	1	0.516	0.484	0.516
BB	7	7	8	16	7	0	16	1	1	0.997	0.003	0.997
BB	7	7	8	16	5	0	16	1	1	0.994	0.006	0.994
BB	12	7	8	16	5	0	16	1	1	0.988	0.012	0.988
BB	12	7	8	16	5	0	16	1	1	0.988	0.012	0.988
BB	12	7	8	16	5	0	16	1	1	0.988	0.012	0.988
BB	7	7	8	16	7	9	16	1	1	0.979	0.021	0.979
BB	7	7	8	16	7	9	16	1	1	0.979	0.021	0.979
BB	10	8	8	16	7	0	16	1	1	0.979	0.021	0.979
BB	7	7	8	10	5	25	16	1	1	0.969	0.031	0.969
BB	10	8	8	16	5	0	16	1	1	0.963	0.037	0.963
BB	10	8	8	16	5	0	16	1	1	0.963	0.037	0.963
BB	10	8	8	16	5	0	16	1	1	0.963	0.037	0.963
BB	12	7	8	16	7	9	16	1	1	0.938	0.062	0.938
BB	12	7	8	16	7	9	16	1	1	0.938	0.062	0.938
BB	7	7	8	16	5	25	16	1	1	0.928	0.072	0.928
BB	7	7	8	16	5	25	16	1	1	0.928	0.072	0.928
BB	7	7	8	16	5	25	16	1	1	0.928	0.072	0.928
BB	7	7	8	16	5	25	16	1	1	0.928	0.072	0.928
BB	10	8	8	10	7	9	16	1	1	0.925	0.075	0.925
BB	10	8	8	10	5	25	16	1	1	0.872	0.128	0.872
BB	6	8	8	16	7	0	16	1	1	0.861	0.139	0.861
BB	6	8	8	16	7	0	16	1	1	0.861	0.139	0.861
BB	6	8	8	16	7	0	16	1	1	0.861	0.139	0.861
BB	12	7	8	16	5	25	16	1	1	0.853	0.147	0.853
BB	12	7	8	16	5	25	16	1	1	0.853	0.147	0.853
BB	10	8	8	16	7	9	16	1	1	0.829	0.171	0.829
BB	6	8	8	16	5	0	16	1	1	0.796	0.204	0.796
BB	6	8	8	16	5	25	16	1	0	0.779	0.779	0.221
BB	6	8	8	16	5	25	16	1	0	0.779	0.779	0.221
BB	6	8	8	16	5	25	16	1	0	0.779	0.779	0.221
BB	6	8	8	16	5	25	16	1	0	0.779	0.779	0.221
BB	10	8	8	16	5	25	16	1	1	0.656	0.344	0.656
BB	10	8	8	16	5	25	16	1	1	0.656	0.344	0.656

BB	10	8	8	16	5	25	16	1	1	0.656	0.344	0.656
BB	10	8	8	16	5	25	16	1	1	0.656	0.344	0.656
BB	6	8	8	16	7	9	16	1	0	0.592	0.592	0.408
BB	6	8	8	16	7	9	16	1	0	0.592	0.592	0.408
BB	6	8	8	16	7	9	16	1	0	0.592	0.592	0.408
BB	6	8	8	16	7	9	16	1	0	0.592	0.592	0.408
BB	6	8	8	16	7	9	16	1	0	0.592	0.592	0.408
